# Characterization of red pigmented yeasts and genes associated with astaxanthin synthesis in *Rhodotorula mucilaginosa* HL26-1 and *Rhodotorula paludigena* LL69-1

**DOI:** 10.12688/f1000research.164600.1

**Published:** 2025-07-21

**Authors:** Patcharaporn Hoondee, Nisachon Tedsree, Sukanya Phuengjayaem, Engkarat Kingkaew, Boonchoo Sritularak, Pornchai Rojsitthisak, Takuji Nakashima, Worathat Thitikornpong, Somboon Tanasupawat

**Affiliations:** 1 Division of Biology, Faculty of Science and Technology, Rajamangala University of Technology Krungthep, Sathon, Bangkok, 10120, Thailand; 2 Biodiversity and Sustainable Utilization Research Unit, Rajamangala University of Technology Krungthep, Sathon, Bangkok, 10120, Thailand; 3 Faculty of Science and Arts, Chanthaburi Campus, Burapha University, Tha Mai, Chanthaburi, 22170, Thailand; 4 Department of Microbiology, Faculty of Science, King Mongkut's University of Technology Thonburi, Thung Khru, Bangkok, 10140, Thailand; 5 Department of Biology, School of Science, King Mongkut's Institute of Technology Ladkrabang, Lat Krabang, Bangkok, 10520, Thailand; 6 Center of Excellence in Natural Products for Ageing and Chronic Diseases, Chulalongkorn University, Pathumwan, Bangkok, 10330, Thailand; 7 Department of Pharmacognosy and Pharmaceutical Botany, Faculty of Pharmaceutical Sciences, Chulalongkorn University, Pathumwan, Bangkok, 10330, Thailand; 8 Department of Food and Pharmaceutical Chemistry, Faculty of Pharmaceutical Sciences, Chulalongkorn University, Pathumwan, Bangkok, 10330, Thailand; 9 Department of Field Sciences, University of Human Environments, Mutsuyama, Ehime, 790-0825, Japan; 10 Research Organization for Nano and Life Innovation, Waseda University, Shinjuku, Tokyo, 162-0041, Japan; 11 Department of Biochemistry and Microbiology, Chulalongkorn University, Pathumwan, Bangkok, 10330, Thailand

**Keywords:** astaxanthin, pigmented yeast, Rhodotorula mucilaginosa, Rhodotorula paludigena, astaxanthin synthase

## Abstract

**Background:**

Astaxanthin, a red xanthophyll carotenoid, is a powerful antioxidant, anticancer, and glucose and lipid homeostasis regulator. Some pigmented yeasts belonging to the genus *Rhodotorula*, the well-known yeast for beta-carotene production, have been reported as natural astaxanthin producers. However, the lack of genomic data on astaxanthin-producing strains within these species hinders the identification of biosynthetic routes, molecular characterization of these pathways, and gene editing applications.

**Methods:**

This study explored the diversity and astaxanthin production capability of cultivable pigmented yeast in flower samples. The astaxanthin production ability was inspected by three consecutive methods, including thin-layer chromatography (TLC) for the preliminary step, followed by quantitative spectrophotometry and high-performance liquid chromatography (HPLC) for qualitative validation. The draft genome sequence and astaxanthin-producing genes of astaxanthin-producing yeasts were examined.

**Results:**

Twelve of 23 yeasts from floral samples exhibited natural pigmentation, with colors ranging from pinkish-orange to red, and exhibited the potential for astaxanthin synthesis. These yeasts were *Rhodotorula paludigena* (three strains) and *Rhodotorula mucilaginosa* (nine strains). Among *R. mucilaginosa* strains, HL26-1 had the greatest astaxanthin content (104.98 ± 0.13 μg/g DCW) and yield (0.9280 ± 0.0012 mg/L). Strain LL69-1 has the greatest astaxanthin content (275.94 ± 0.16 μg/g DCW) and yield (1.8632 ± 0.0023 mg/L) among *R. paludigena* strains. The 18.78 Mbp *R. mucilaginosa* HL26-1 genome includes 5,711 protein-coding genes. Conversely, the *R. paludigena* LL69-1 genome was 20.99 Mbp, encompassing 6,782 predicted genes. A comprehensive investigation of draft genome sequences of these two strains identified *CrtE*, *CrtYB*, *CrtI*, *CrtS*, and *CrtR* as potential astaxanthin transcription genes.

**Conclusion:**

Here, our results highlight the outstanding potential of two naturally pigmented yeasts, *R. mucilaginosa* HL26-1 and *R. paludigena* LL69-1, for astaxanthin production. Furthermore, our findings provide information on the whole genome and protein-encoded genes associated with astaxanthin production, which serve as valuable biological resources for various biotechnological applications.

## Introduction

Astaxanthin (3,3′-dihydroxy-β, β′-carotene-4,4′-dione), a red compound belonging to the xanthophyll carotenoid group, exhibits formidable antioxidant capabilities, surpassing those of vitamin E (alpha-tocopherol), β-carotene, canthaxanthin, and other natural carotenoids by a factor of 100. ^1^ Astaxanthin is widely used in various industries, including beauty, food, animal feed, health supplements, and pharmaceuticals. ^2^ Astaxanthin serves as a potent metabolic regulator of glucose and lipid homeostasis. Their functions include increased insulin sensitivity, augmented glucose uptake, enhanced lipid turnover, and diminished lipid synthesis in the liver. Moreover, astaxanthin has garnered substantial attention in dermatology because of its ability to counteract oxidative stress, facilitate cellular rejuvenation, repair DNA damage, and protect against UV-induced photoaging and skin malignancies. ^3, 4^ Furthermore, astaxanthin shows its anticancer properties, attributable to its ability to scavenge free radicals and quell singlet oxygen. ^5^

The acquisition of astaxanthin and other pigments involves two primary methods: synthetic production utilizing chemicals and natural extraction. ^6^ Despite its cost-effectiveness compared to natural sources, chemical pigment manufacturing can generate hazardous byproducts and pose environmental risks. ^7^ Natural astaxanthin has been identified in diverse organisms, including shrimp, lobsters, fish, crustaceans, salmon, trout, red sea breams, and microbes. ^8^ Among microorganisms, *Haematococcus pluvialis* has stood out for decades because of its superior bioactivity compared to that of synthetically produced astaxanthin. However, challenges in both upstream and downstream processes have made the production of algal astaxanthin economically and logistically challenging. ^2^ Therefore, several studies focused on evaluating the natural astaxanthin-producing ability of other potential microorganisms, particularly the pigmented yeast, in order to realize commercial astaxanthin production. Compared to microalgae, yeast cultivation can be cultured in bioreactors, utilize various carbon sources from lignocellulosic biomass and industrial by-products, and reduce land management and environmental concerns. ^6, 9– 11^

*Phaffia rhodozyma* ( *Xanthophyllomyces dendrorhous*) is the rare yeast species with current biotechnological use in the production of astaxanthin for several decades. ^6, 12^ Recently, it has been shown that some *Rhodotorula* species, which were previously known for their ability to synthesize beta-carotene, might be used as an alternative for astaxanthin such as *Rhodotorula toruloides,* ^13, 14^ *Rhodotorula paludigena,* ^15, 16^ *Rhodotorula mucilaginosa,* ^17^ and *Rhodotorula* sp. ^18^ The pathway of astaxanthin biosynthetic in *Phaffia rhodozyma* has been reported. ^19– 21^ The regulation starts with beta-carotene formation through mevalonate pathway. Acetyl-CoA was converted to isopentenyl-pyrophosphate (IPP), the precursor of all isoprenoids, which were further condensed to produce phytoene, the colorless carotenoid. Subsequently, phytoene was transformed into β-carotene, which was catalyzed by phytoene synthase/lycopene beta-cyclase and phytoene desaturase (encoded by *CrtYB* and *CrtI* genes, respectively). ^19– 21^ Finally, a single gene called *CrtS*, which could act as both a ketolase and a hydroxylase, was responsible for converting β-carotene into astaxanthin. ^20, 21^ Nevertheless, the astaxanthin biosynthesis pathway in *Rhodotorula* species remain limited. Unfortunately, the lack of genomic information on astaxanthin-producing within these genera hampers the discovery of biosynthetic pathways and their molecular characterization and gene editing applications. Only a handful of studies have employed effective techniques, such as whole-genome sequencing and gene analysis, to elucidate genome-related astaxanthin production in *Rhodotorula* yeast. ^13, 15, 16, 18^

This study aimed to isolate and characterize novel astaxanthin-producing yeast strains obtained from flowers. Furthermore, we investigated the genomic profiles of two chosen astaxanthin-producing yeast strains, namely, *Rhodotorula mucilaginosa* HL26-1 and *Rhodotorula paludigena* LL69-1. Through analysis of their whole-genome sequences and identification of genes associated with astaxanthin synthesis, our objective was to provide comprehensive insights into the repertoire of proteins encoded within their genomes. This endeavor holds promise for elucidating their functional capabilities and igniting excitement about their potential integration as valuable biological resources across a spectrum of biotechnological applications, from the food and feed industries to the pharmaceutical and cosmetic sectors.

## Methods

### Isolation and phenotypic characterization

We collected eleven flower samples from the residential areas in Lampang Province, Thailand, in April 2022. Flower identification was conducted on-site by interviewing the owner of the plant and was then identified by one of the co-authors (B.S.). The samples were carefully transported to the laboratory in sterile plastic bags with ice packs to maintain integrity during transit. Specimens have been deposited at the Department of Pharmacognosy and Pharmaceutical Botany, Faculty of Pharmaceutical Sciences, Chulalongkorn University, Thailand. Detailed information regarding the samples, specimen number, and their respective collection locations is provided in Table S1. Each sample was inoculated into 15 mL of yeast malt (YM) medium containing 10 g/L glucose, 5 g/L peptone, 3 g/L yeast extract, and 3 g/L malt extract at pH 5.5. The inoculated media were incubated at 25°C for 3 days. Subsequently, the resulting cultures were streaked onto YM agar medium containing 20 g/L agar, 10 g/L glucose, 5 g/L peptone, 3 g/L yeast extract, and 3 g/L malt extract (pH 5.5) and incubated at 30°C for 5 days. Following incubation, pigmented yeast colonies displaying the desired pink to red hue were meticulously selected and purified for clonality on YM agar media using the streak plate method. Each clonal culture was carefully preserved on a YM agar slant at 4°C to facilitate further analysis and experimentation. Carbon assimilation of strain was performed using the API ^®^ ID 32 C kit (BioMérieux, France) according to the manufacturer’s instructions. Reactions were visually examined at 72 h, and then the results were interpreted to be positive or negative based on the presence or absence of turbidity in the carbohydrate wells.

### Molecular identification of yeast strains

*Genomic DNA extraction*

Genomic DNA was extracted from the pure yeast cultures using the glass bead extraction method ^22^ with some modification. Briefly, yeast cells were washed twice with sterilized distilled water and then lysed by vortexing with 0.3 g of ∅ 0.45-0.52 mm acid washed glass beads in 200 μL of extraction buffer (comprising 2% (v/v) Triton X-100, 1% (w/v) SDS, 100 mM NaCl, 10 mM Tris-HCl (pH 8.0), and 1 mM EDTA (pH 8.0)) for 5 minutes. The resulting cell-free supernatant was transferred to a new tube and gently mixed with a 2X volume of phenol (Sigma–Aldrich ^®^, USA, Cat. No. 242322), chloroform (RCI Labscan ^®^, Ireland, Cat. No. AR 1027E), and isoamyl alcohol (Sigma–Aldrich ^®^, USA, Cat. No. 8.22255) at a ratio of 25:24:1. After centrifugation, the supernatant was transferred to a new tube containing 1 mL of absolute ethanol (RCI Labscan ^®^, Ireland, Cat. No. AR1069) and stored at -20°C for 20 minutes. The DNA pellet obtained after centrifugation was washed with 500 μL of 70% (v/v) ethanol, dried at 37°C, dissolved in 50 μL of TE buffer, and stored at -20°C until further use.

*Sequencing of the 26S rRNA gene (D1/D2 domain)*

The 26S rRNA gene within the D1/D2 domain of the large subunit (LSU D1/D2 domain) was amplified by PCR using the primers NL1 (5′-GCATATCAATAAGCGGAGGAAAAG-3′) and NL4 (5′-GGTCCGTGTTTCAAGACGG-3′). ^23^ PCR was conducted in a 20 μL volume comprising 2 μL of DNA template, 0.4 μL of each primer (10 pmol/μL), 10 μL of 2X Go Tag Green, and 7.2 μL of distilled water. The amplification process involved an initial denaturation step at 94°C for 3 minutes, followed by 36 cycles of denaturation at 94°C, annealing at 52°C, and extension at 72°C, each for 30 seconds, and a final extension step at 72°C for 5 minutes. Subsequently, the PCR products were purified using a gel/PCR DNA fragment extraction kit (GenepHlow™, Geneaid Biotech Ltd., Taiwan). Purified PCR products were sequenced bidirectionally using BT sequencing technology (Celemics Inc., Republic of Korea).

*Analysis of phylogenetic placement*

The LSU D1/D2 sequences were aligned with those of related species using MUSCLE, ^24^ and any gaps were removed. MEGA11 software ^25^ was used to construct a neighbor-joining (NJ) tree using Kimura’s two-parameter model. ^26^ The reliability of the branches was assessed using a bootstrap test with 1000 replicates. ^27^

### Yeast cultivation for astaxanthin production

A single loopful of yeast cultured on YM agar was transferred to YM broth (50 mL) in a 250 mL flask and then incubated at 30°C with agitation at 200 rpm for 24 hours. Subsequently, a 5 mL aliquot of this culture was inoculated into fresh YM broth (45 mL) in a 250 mL flask and incubated under the same conditions for 72 hours. Following incubation, the cells were harvested by centrifugation at 4°C and 6,500 × g, for 10 minutes, washed twice with distilled water, and then subjected to lyophilization for drying. The resultant dry cell weight (DCW) was determined to quantify the cell biomass. Lyophilized cells were used for further analysis of astaxanthin.

### Astaxanthin analysis

*Qualitative analysis of astaxanthin production by thin layer chromatography*

The extraction and qualitative analysis of astaxanthin were performed using a slightly modified method described by Ushakumari and Ramanujan. ^28^ Lyophilized cells (0.01 g DCW) were suspended in 1 mL of acetone (RCI Labscan ^®^, Ireland, Cat. No. AR1003) and homogenized using a pestle at room temperature for 3 min. The supernatant was collected by centrifugation at 12,300 × g, for 10 minutes. Subsequently, a 20 μL aliquot of the extracted sample was applied to a TLC silica gel G plate using a capillary along with standard astaxanthin (DYCN ^®^, Dayang Chem Co. Ltd., China). A mixture of acetone (RCI Labscan ^®^, Ireland, Cat. No. AR1003) and hexane (RCI Labscan ^®^, Ireland, Cat. No. AR1085) was used as the mobile phase for spot development at a ratio of 1:3 (v/v). The colored bands were directly observed after development and compared with the standard astaxanthin band under visible light. In addition, the retention factor (R _f_) value was calculated to quantify the movement of the materials along the plate.

*Quantification of astaxanthin production*

The extraction and measurement of astaxanthin were conducted using previously described methods with slight modifications. ^29^ To extract the intracellular carotenoid content, 50 mg of lyophilized cells were suspended in 5 mL of DMSO (Sigma–Aldrich ^®^, USA, Cat. No. 34869), ultrasonicated at 37 kHz and 50°C for 30 min (Elmasonic, E60H model, Germany). The resulting cell extracts were centrifuged at 12,300 × g, for 5 minutes. The extraction process was repeated until the supernatant became colorless. The astaxanthin concentration was determined using a Cary 60 UV–Vis spectrophotometer (Agilent) at 530 nm against a pure DMSO blank. ^29, 30^ A standard curve of absorbance versus astaxanthin concentration was generated using the following concentrations: 0, 0.25, 0.5, 1, 2, 4, 6, and 8 μg/mL in DMSO. Astaxanthin concentrations were calculated using the standard calibration curve of AXs. The results are presented as the mean of triplicate measurements.

*Confirmative analysis of astaxanthin synthesis*

The astaxanthin was extracted and analyzed based on the described method. ^15^ In brief, 0.05 g of dried yeast cell and 5 mL of DMSO (Sigma–Aldrich ^®^, USA, Cat. No. 34869) were mixed and sonicated at 55°C, 5 min with 37 kHz of sonication (Elmasonic E60H, Germany). After centrifugation at 6,500 × g for 10 min, the resultant supernatant was filtered (0.22 μm) and then was transferred to chromatography column. Astaxanthin were analyzed by high performance liquid chromatography (HPLC) using UHPLC Nexera X2 system (Shimadzu, Japan) consisting of LC-30AD binary pump with a CTO-20AC column oven, an SPD-M30A detector and an SIL-30AC autosampler. Astaxanthin were separated using C18 column (GL Science InterSustain, 4.6 mm × 150 mm, 5 μm) using mobile phases consisting of mixture of methanol (RCI Labscan ^®^, Ireland, Cat. No. LC1115)/acetonitrile (RCI Labscan ^®^, Ireland, Cat. No. LC1005)/ethyl acetate (RCI Labscan ^®^, Ireland, Cat. No. LC1070) /formic acid (Sigma–Aldrich ^®^, USA, Cat. No. 5.43804) (75.9:12:12:0.1, v/v) and methanol. Analytical-grade astaxanthin was purchased from Dayang Chem Co. Ltd. (China) and utilized as a reference standard.

### Whole-genome sequencing and analysis

The whole genome was sequenced using the paired-end (PE) 150 method on the Illumina HiSeq Xten/Novaseq/MGI2000 platform at Vishuo Biomedical Pte. Ltd., Beijing, China. The single-end reads were processed to eliminate adapters and low-quality bases using Fastp (v0.23.0). The resulting data were then assembled into contigs using Velvet de Novo assembler version 1.2.10. ^31, 32^ Subsequently, the contigs were assembled into scaffolds using SSPACE (version 3.0), ^33^ and the gaps were filled using GapFiller (versions 1–10). ^34^ Gene prediction was performed using Augustus version 3.3. ^35^ The coding genes were annotated using the National Center for Biotechnology Information (NCBI) NR database via BLAST. Subsequently, gene function prediction was carried out using the Kyoto Encyclopedia of Genes and Genomes (KEGG) ^36^ and KofamKOALA tools, employing default settings and considering all hits ( https://www.genome.jp/kegg/). ^37^ Proksee ( https://proksee.ca/) was used to generate the circular genome map, and OrthoVenn ( https://orthovenn3.bioinfotoolkits.net/ start/db) was used to create the Venn diagram. The Kostas Lab web-based tool ( http://enve-omics.ce.gatech.edu/) was used to analyze the average nucleotide identity (ANI) values. ^38^

## Results

### Isolation and identification of yeast strains

Twenty-three yeasts were isolated from the 11 flower samples collected (Table S1). Among them, 12 isolates exhibited pigmented colonies with the desired coloration ranging from pink to orange. All isolates were subjected to identification at the molecular operational taxonomic unit (MOTU) level by sequencing of the 26S rRNA gene (LSU D1/D2 domain), followed by species assignment via comparison with entries in the NCBI GenBank database using the BLASTn program. Consequently, all 12 pigment yeast strains were identified as basidiomycetous yeasts (Table S1), with two species belonging to the *Rhodotorula* genus: *Rhodotorula paludigena* (9 strains) and *Rhodotorula mucilaginosa* (3 strains). The remaining 11 strains were classified into four species of Basidiomycota— *Cryptococcus heveanensis* (1 strain), *Pseudozyma aphidis* (2 strains), *Pseudozyma hubeiensis* (1 strain), and *Pseudozyma siamensis* (1 strain)—and five species of Ascomycota— *Candida parapsilosis* (1 strain), *Metschnikowia koreensis* (1 strain), *Saccharomyces cerevisiae* (2 strains), *Wickerhamiella infanticola* (1 strain), and *Debaryomyces nepalensis* (1 strain).

### Qualitative analysis of astaxanthin production by thin layer chromatography

**Figure 1.**
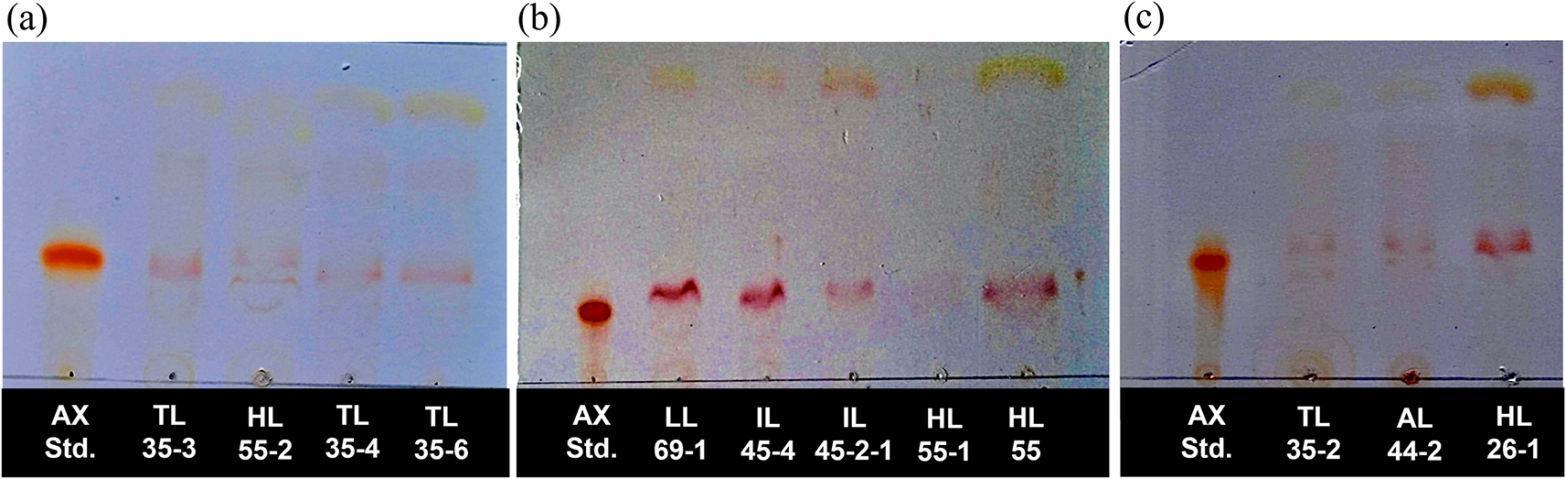
Thin layer chromatography (TLC) of an acetone extract of pigment isolated yeasts and astaxanthin standard on Silica gel G plate using a mixture of acetone and hexane (ratio of 1:3 (v/v) as the mobile phase. The presence of astaxanthin band of *R. paludigena* strain TL35-3, HL55-2, TL35-4, and TL35-6 (a), strain LL69-1, IL45-1, IL45-2-1, HL55-1, HL55 (b), and *R. mucilaginosa* strain TL35-2, AL44-2, and HL26-1 (c).

A total of twelve pigmented strains were selected in order to conduct a qualitative examination of their astaxanthin synthesis by using the thin-layer chromatography (TLC) technique. Astaxanthin, which is red, requires no additional processing for spot visualization. Compared with that of the astaxanthin standard, an astaxanthin band was observed in all extracts, with a R _f_ value of 0.28. The experimental results demonstrated the presence of an astaxanthin band in all tested yeast strains ( Figure 1). All yeast strains were selected for further quantitative analysis of astaxanthin content.

### Quantification of astaxanthin production

**Figure 2.**
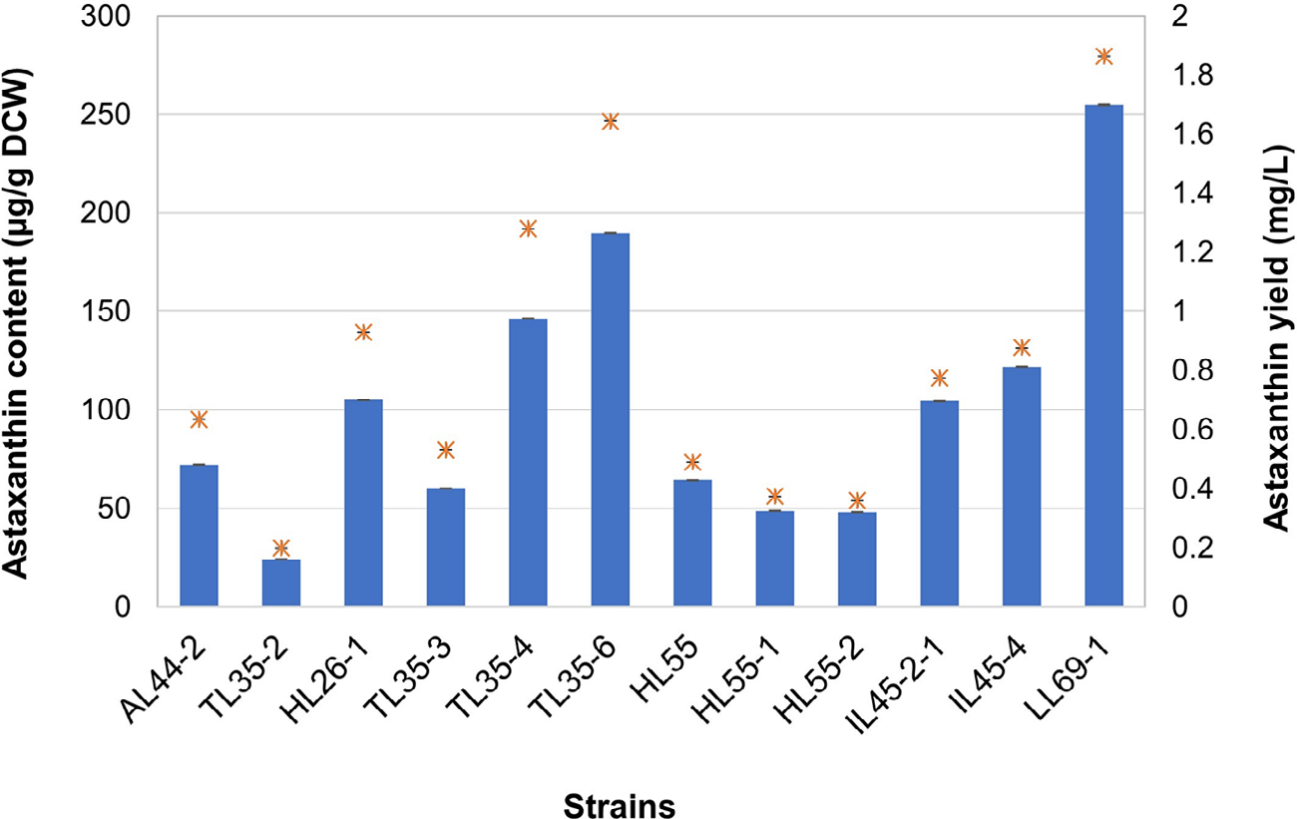
Quantitative analysis of astaxanthin production by 3 *R. mucilaginosa* strains (AL44-2, TL35-2, and HL26-1) and 9 *R. paludigena* strains (TL35-5, TL35-4, TL35-6, HL55, HL55-1, HL55-2, IL45-2-1, IL45-4, and LL69-1). The results are reported as the average value ± standard deviation (SD) calculated from three repeated measurements.

Twelve yeast strains exhibiting positive results in TLC analysis for astaxanthin were subjected to quantitative analysis using spectrophotometry. Intracellular astaxanthin was extracted from powdery cells using the conventional DMSO extraction method ^29^ coupled with sound energy via an ultrasonic bath to enhance cell disruption efficiency. ^39^ In the *R. mucilaginosa* strains, the astaxanthin content ranged from 23.99 ± 0.22 to 104.98 ± 0.13 μg/g DCW, with yields ranging from 0.1991 ± 0.0021 to 0.9280 ± 0.0012 mg/L ( Figure 2). Moreover, several *R. paludigena* strains exhibited astaxanthin contents and yields ranging from 47.99 ± 0.11 to 254.78 ± 0.27 μg/g DCW and from 0.3589 ± 0.0009 to 1.8632 ± 0.0023 mg/L, respectively ( Figure 2). Strain LL69-1 demonstrated the highest astaxanthin production of *R. paludigena*, with an astaxanthin content of 254.78 ± 0.27 μg/g DCW and a yield of 1.8632 ± 0.0023 mg/L. Meanwhile, *R. mucilaginosa* strain HL26-1, the highest producer of its species, which yielded 104.98 ± 0.13 μg/g DCW and 0.9280 ± 0.0012 mg/L.

### Confirmative analysis of astaxanthin synthesis in selected astaxanthin-producing strains

The ability of two astaxanthin-producing yeasts, *R. mucilaginosa* HL26-1 and *R. paludigena* LL69-1, to synthesize astaxanthin was confirmed by high-performance liquid chromatography (HPLC). The HPLC chromatogram displayed astaxanthin peaks from strains HL26-1 and LL69-1, along with a 10 ppm astaxanthin standard. The retention time of the standard peak was used as a reference, confirming the presence of astaxanthin in the strain HL26-1 and LL69-1 with accuracy. The highest points within the time intervals during which HL26-1 and LL69-1 were retained were observed at 2.061 and 2.211 minutes, respectively. These findings provided conclusive evidence that both strains, HL26-1 and LL69-1, effectively synthesized astaxanthin (Supplementary Figure S1).

### General features of the *R. paludigena* LL69-1 and *R. mucilaginosa* HL26-1 genomes

**Figure 3.**
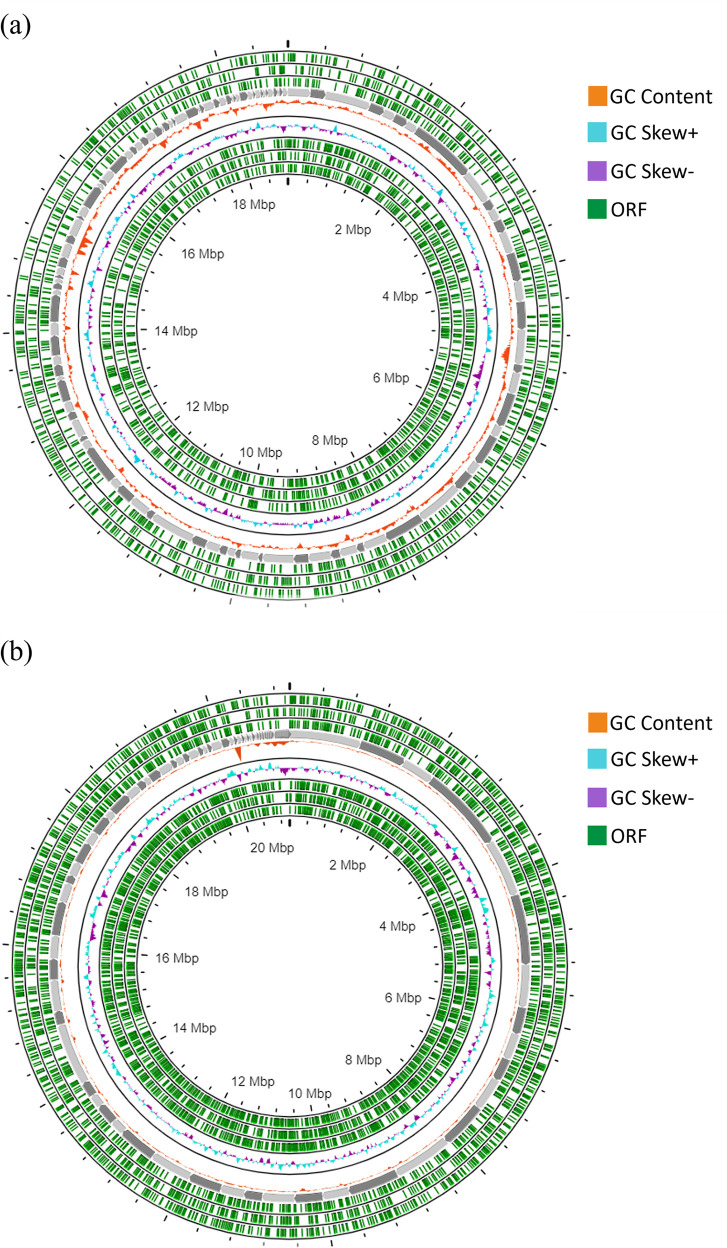
Circular genomic map of *R. mucilaginosa* HL26-1 and *R. paludigena* LL69-1 displaying the following information: GC content in orange, GC skew (+) in blue, GC skew (-) in purple, and open reading frames (ORFs) in green.

The genome assembly statistics for the two astaxanthin-producing yeasts, *R. mucilaginosa* HL26-1 and *R. paludigena* LL69-1, are presented in Table 1. The assembly of *R. mucilaginosa* HL26-1 resulted in 154 scaffolds, with the giant scaffold spanning 1,230,191 base pairs (bp). The N50 length of the scaffolds was 476,521 bp, with a GC content of 63.82% and a genome size of 20.99 Mbp. Conversely, *R. paludigena* LL69-1 had a genome size of 18.78 Mbp, with a GC content of 60.12%. Its assembly comprised 107 scaffolds with an N50 value of 254,092 bp and an L50 value of 24 scaffolds. The largest scaffold in LL69-1 was 853,611 bp, whereas the shortest was 329 bp. Circular genomics of the HL26-1 and LL69-1 genomes, illustrating the open reading frame (ORF) positions, GC content, and GC skew of each strain, are depicted in Figure 3. The whole-genome sequences of *R. mucilaginosa* HL26-1 and *R. paludigena* LL69-1 were deposited in the NCBI/GenBank database ( http://www.ncbi.nlm.nih.gov), associated with the BioProject identities PRJNA1025132 and PRJNA1025134, BioSample numbers SAMN37714267 and SAMN37714269, and GenBank accession numbers JAZBNE000000000 and JAWJBI000000000, respectively.

**Table 1.** Assembly statistics of the *R. paludigena* HL26-1 and *R. mucilaginosa* LL69-1 genomes.

Features	Strains	
	*R. mucilaginosa* HL26-1	*R. paludigena* LL69-1
Genome assembly size (bp)	18,775,076	20,987,037
Max length (bp)	853,611	1,230,191
Number of scaffolds	107	154
N50 (bp)	193,129	476,521
GC (%)	60.12	63.82
Predicted protein-coding gene	5,711	6,782

### Phylogenetic and average nucleotide identity (ANI) analysis of strains

Figure S2 depicts a phylogenetic tree using the D1/D2 domains of the large subunit sequences obtained in this study and those available in the NCBI nucleotide database. This analysis confirmed the phylogenetic relationship between these pigmented yeasts and other related species. *R. mucilaginosa* HL26-1 and *R. paludigena* LL69-1 were clustered within the same clade as their closest-type strains, *R. mucilaginosa* CBS316 ^T^ and *R. paludigena* CBS6566 ^T^, respectively, with 100% bootstrap support.

Moreover, we conducted an average nucleotide identity (ANI) analysis, a crucial step in assessing the phylogenomic relationships between the two astaxanthin-producing yeasts and other *Rhodotorula* species. The pairwise ANI values of the whole genomes of *R. mucilaginosa* HL26-1, *R. paludigena* LL69-1, and the other *Rhodotorula* species varied (Supplementary Figure S3).

### Functional genome annotation and comparative analysis of putative gene families associated with astaxanthin biosynthesis

**Figure 4.**
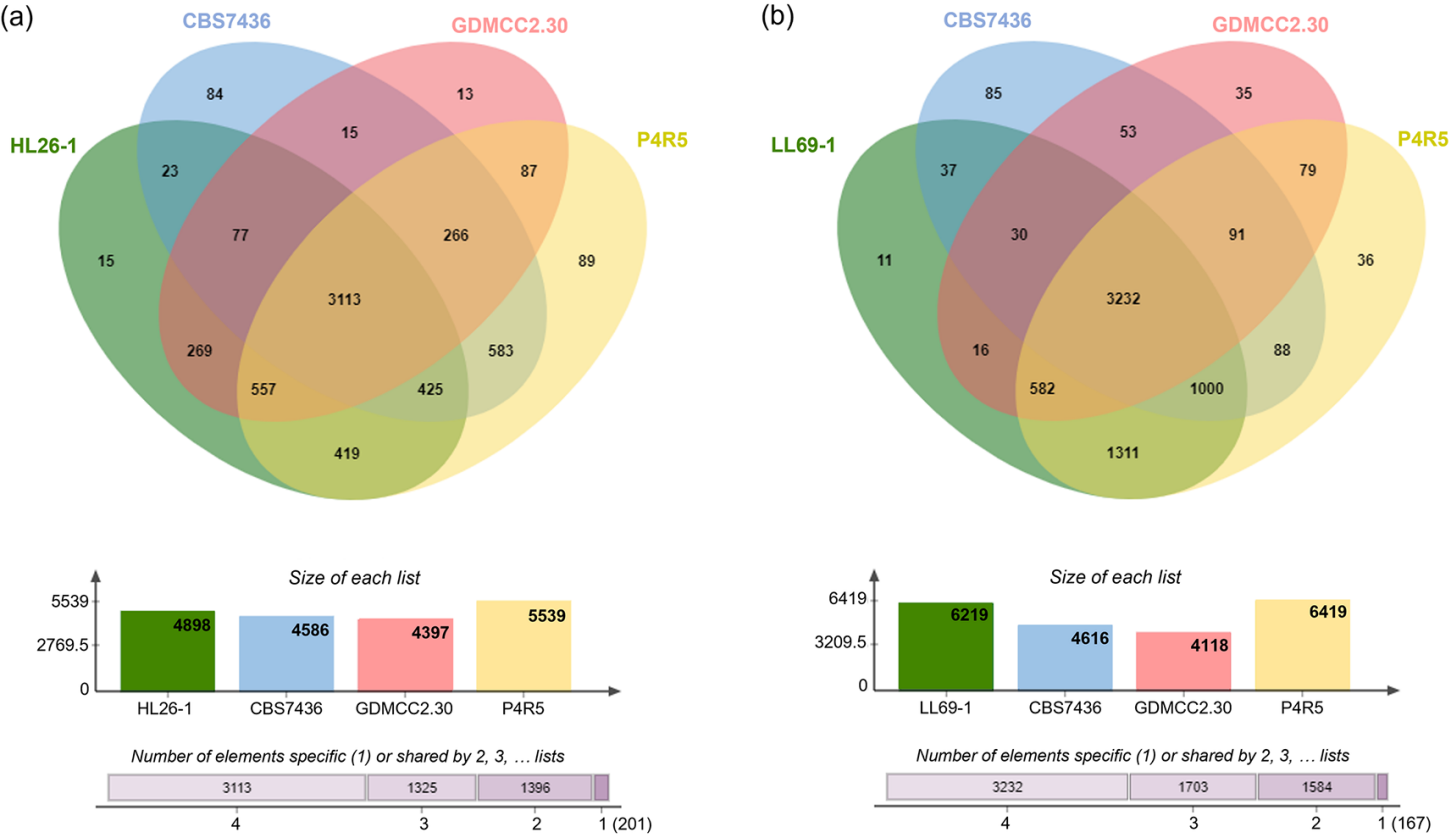
Venn diagram displaying the presence of conserved and specific proteins among *Rhodotorula mucilaginosa* HL26-1 (a) or *R. paludigena* LL69-1 (b) and *R. kratochvilovae* CBS 7436, *R. mucilaginosa* GDMCC2.30, and *R. paludigena* P4R5.

According to annotation results from the National Center for Biotechnology Information (NCBI) database, the draft genome sequence of *R. mucilaginosa* HL26-1 comprises 5,711 protein-encoding genes, while *R. paludigena* LL69-1 was predicted to contain approximately 6,782 coding genes. The Venn diagram in Figure 4 illustrates the genetic variations and distinct characteristics that differentiate the genomes of HL26-1 and LL69-1 from those of closely related species. The protein-coding sequences of HL26-1 and LL69-1, along with those of three other *Rhodotorula* species ( *R. kratochvilovae* CBS 7436, *R. mucilaginosa* GDMCC2.30, and *R. paludigena* P4R5), were compared to examine the similarity of their protein sequences. This comparison revealed that *R. mucilaginosa* HL26-1, *R. kratochvilovae* CBS 7436 *, R. mucilaginosa* GDMCC2.30, and *R. paludigena* P4R5 possess 15, 84, 13, and 89 proteins, respectively, which are exclusive to their respective species or strains. Additionally, 11, 85, 35, and 36 protein families were identified as being species- or strain-specific for *R. paludigena* LL69-1, *R. kratochvilovae* CBS 7436 ^T^ *, R. mucilaginosa* GDMCC2.30, and *R. paludigena* P4R, respectively.

KEGG metabolic pathway analysis categorized a total of 2,795 (52.1%) protein-coding genes of HL26-1 into four major groups: metabolism (951 genes), genetic information processing (682 genes), cellular activities (225 genes), and environmental information processing (137 genes). Within the LL69-1 dataset, 3,303 protein-coding genes (48.7%) were subjected to KEGG analysis and classified into four major categories: metabolism (1,064 genes), genetic information processing (734 genes), cellular activities (245 genes), and environmental information processing (168 genes).

The putative gene families associated with astaxanthin biosynthesis in strains HL26-1 and LL69-1 were determined ( Table 2). The pathway of astaxanthin synthesis was divided into two main sections: one that creates the basic building blocks called terpenoids, which are important natural substances, and another that specifically produces astaxanthin.

**Table 2.** Putative gene families associated with astaxanthin biosynthesis in *R. mucilaginosa* HL26-1 and *R. paludigena* LL69-1.

Pathways	Putative genes	Enzyme product (KEGG orthologs number, EC number)	LL69-1		HL26-1	
			Scaffold ID	E-value	Scaffold ID	E-value
Terpenoid backbone biosynthesis	*ACAT*	Acetyl-CoA acetyltransferase (K00626, EC:2.3.1.9)	Scaffold13.g2648	2.1×10 ^−168^	Scaffold38.g4472	5.1×10 ^−172^
	*HMGCS*	Hydroxymethylglutaryl-CoA synthase (K01641, EC:2.3.3.10)	Scaffold18.g3608	7.1×10 ^−197^	Scaffold33.g3151	6.4×10 ^−194^
	*HMGCR*	Hydroxymethylglutaryl-CoA reductase (K00021, EC:1.1.1.34)	Scaffole80.g6723	3.0×10 ^−296^	Scaffold61.g2990	9.1×10 ^−298^
	*MVD*	Mevalonate kinase (K00869, EC:2.7.1.36)	Scaffold13.g2759	2.5×10 ^−108^	Scaffold39.g1654	7.3×10 ^−103^
	*PMK*	Phosphomevalonate kinase (K00938, EC:2.7.4.2)	Scaffold13.g2880	7.2×10 ^−122^	Scaffold38.g4446	1.0×10 ^−121^
	*DPMDC*	Diphosphomevalonate decarboxylase (K01597, EC:4.1.1.33)	Scaffold58.g6593	2.1×10 ^−150^	Undetectable	-
	*IDI*	Isopentenyl-diphosphate delta isomerase (K01823, EC:5.3.3.2)	Scaffold13.g2706	1.5×10 ^−67^	Scaffold39.g1606	8.8×10 ^−68^
	*GGDPS*	Geranylgeranyl diphosphate synthase (K00804, EC:2.5.1.1 2.5.1.10 2.5.1.29)	Scaffold4.g827	1.0×10 ^−149^	Scaffold103.g709	5.6×10 ^−148^
	*FDPS*	Farnesyl diphosphate synthase (K00787, EC:2.5.1.1 2.5.1.10)	Scaffold14.g3047	1.1×10 ^−154^	Scaffold77.g4986	1.3×10 ^−155^
	*CrtE*	Geranylgeranyl pyrophosphate synthase (K05355, EC:2.5.1.82 2.5.1.83)	Scaffold1.g227	2.2×10 ^−168^	Scaffold17.g303	7.0×10 ^−171^
Astaxanthin biosynthesis	*CrtYB*	Phytoene synthase/lycopene beta-cyclase (K17841, EC:2.5.1.32 5.5.1.19)	Scaffold3.g680	7.9×10 ^−183^	Scaffold38.g4383	8.5×10 ^−170^
	*CrtI*	Phytoene desaturase (K15745, EC:1.3.99.30)	Scaffold3.g682	2.8×10 ^−234^	Scaffold38.g4386	9.8×10 ^−237^
	*CrtS*	Beta-carotene 4-ketolase/3-hydroxylase (K23037, EC:1.14.99.63 1.14.15.24 1.14.99.-)	Scaffold27.g4592	3.5×10 ^−81^	Scaffold49.g3491	6.2×10 ^−73^
	*CrtR*	Cytochrome P450 (K14338, EC:1.14.14.1 1.6.2.4)	Scaffold6.g1523	8.4×10 ^−68^	Scaffold42.g2465	2.9×10 ^−70^

The key genes associated with terpenoid biosynthesis discovered in the genomes of both strains were *ACAT* (acetyl-CoA acetyltransferase), *HMGCS* (hydroxymethylglutaryl-CoA synthase), *HMGCR* (hydroxymethylglutaryl-CoA reductase), *MVD* (mevalonate kinase), *PMK* (phosphomevalonate kinase), *IDI* (isopentenyl-diphosphate delta isomerase), *GGDPS* (geranylgeranyl diphosphate synthase), *FDPS* (farnesyl diphosphate synthase), and *CrtE* (geranylgeranyl pyrophosphate synthase). The diphosphomevalonate decarboxylase ( *DPMDC*) gene has only been found in strain LL69-1. The multiple gene involved in astaxanthin biosynthesis were annotated in the genomes of both strains, including the *CrtYB* (phytoene synthase/lycopene beta-cyclase), *CrtI* (phytoene desaturase), *CrtS* (beta-carotene 4-ketolase/3-hydroxylase), and *CrtR* (cytochrome P450) genes.

## Discussion

The pigmented yeast in the genus *Rhodotorula* is well established in yeast biotechnology applications and holds promise in numerous industrial sectors, including biofuels, carotenoids, enzymes, bioremediation, cosmetics, and biocontrol agents. ^40– 43^ Recently, some *Rhodotorula* species have exhibited unique abilities to naturally generate astaxanthin, a red pigment with excellent antioxidant activity. These species include *Rhodotorula paludigena* SP9-15, ^15^ *R. paludigena* TL35-5, ^16^ *Rhodotorula sampaioana* PL61-2, ^16^ *Rhodotorula toruloides* VN1, ^13, 14^ and *Rhodotorula* sp. CP72-2. ^18^ Several *Rhodotorula* species has been isolated in this study, the most prevalent was *R. paludigena* (75%), which was found in the African marigold, pink west Indian jasmine, yellow hibiscus, and lantana flower samples. The remaining three *R. mucilaginosa* strains were isolated from flower samples of yellow west Indian jasmine, marigold, and hydrangea. The *Rhodotorula* genus is widespread and commonly found in diverse natural habitats, such as air, soil, lake water, seawater, plants, decomposing plant matter, and various food products and fruit juices. ^44– 49^

Three investigation techniques were implemented to assess the capacity to generate astaxanthin. In the prescreening step, the TLC technique is considered reliable and accurate. Additionally, several studies have widely used TLC to confirm the presence of astaxanthin. ^50– 52^ For the specific quantification of astaxanthin, spectrophotometry has been used. ^29, 30, 53^ Meanwhile, high-performance liquid chromatography (HPLC) has been used as a qualitative confirmative analysis technique for astaxanthin synthesis. All twelve *Rhodotorula* strains identified in this study had the capability to synthesize astaxanthin, a distinctive and uncommon trait among this genus. Nine strains of *R. paludigena* exhibited astaxanthin contents ranging from 47.99 ± 0.11 to 254.78 ± 0.27 μg/g DCW and yields ranging from 0.3589 ± 0.0009 to 1.8632 ± 0.0023 mg/L, respectively. Meanwhile, the astaxanthin content was between 23.99 ± 0.22 and 104.98 ± 0.13 μg/g DCW, and the yields were between 0.1991 ± 0.0021 and 0.9280 ± 0.0012 mg/L for three *R. mucilaginosa* strains. Notably *, R. paludigena* LL69-1 exhibited the highest astaxanthin production, with an astaxanthin content and yield of 254.78 ± 0.27 μg/g DCW and 1.8632 ± 0.0023 mg/L, respectively, surpassing strain HL26-1, the highest producer of the *R. mucilaginosa* species, which yielded 104.98 ± 0.13 μg/g DCW of astaxanthin and 0.9280 ± 0.0012 mg/L of yield. These findings suggest that the ability to produce astaxanthin is species independent, as varying results have been obtained from diverse strains of the same species.

A comparison of the astaxanthin yield (mg/L) among our strains, particularly *R. paludigena* LL69-1, which produced the highest astaxanthin yield (1.86 mg/L, compared with that of other microorganisms), revealed the superior performance of LL69-1. Its astaxanthin yield surpassed that of other wild yeast strains, such as *Phaffia rhodozyma*, which produces 0.2-0.4 mg/L ^54^ and *R. toruloides*, producing 0.93 mg/L. ^55^ However, the astaxanthin production of LL69-1 remains lower than that of many genetically modified strains grown under optimized conditions. ^56, 57^ Likewise, the astaxanthin yields generated by LL 69-1 were significantly lower compared to those produced by the other natural yeast strain and modified strain under optimal conditions. The highest astaxanthin yield of *Phaffia rhodozyma* 7B12 (originated from *P. rhodozyma* Past-1) was 7.71 mg/L when cultivated in optimal nitrogen medium consisting of 0.28 g/L (NH _4_) _2_SO _4_, 0.49 g/L KNO _3_, and 1.19 g/L beef extract. ^56^ The wild strain *Xanthophyllomyces dendrorhous* TISTR 5730 grown in mustard waste precipitated hydrolysate (MPH) under optimal conditions gave the astaxanthin yield of 25.8 mg/L. ^57^ The *X. dendrorhous* strain DW6 produced 374.3 mg/L of astaxanthin, which is the highest amount produced from cane molasses using a two-stage pH method. ^58^ Under optimal conditions, *Rhodotorula* sp. CP72-2 had the highest astaxanthin yield of 4.13 mg/L. ^18^ Meanwhile, the highest yield of *Rhodotorula paludigena* SP9-15 astaxanthin grown in optimized medium and environmental factors was 6.67 mg/L. ^15^ Hence, optimizing culture conditions is crucial for enhancing astaxanthin production. Additionally, there is a need for more valuable genetic data and information on the metabolic pathways associated with astaxanthin synthesis in *R. paludigena* and *R. mucilaginosa.*

The genomic information of two recently obtained astaxanthin-producing yeasts, *R. paludigena* LL69-1 and *R. mucilaginosa* HL26-1, is presented here. The genome sizes of *R. paludigena* LL69-1 and *R. mucilaginosa* HL26-1 were 18.78 Mbp with a GC content of 60.12% and 20.99 Mbp with a GC content of 63.82%, respectively. These findings align with the genome sizes of other pigmented yeast species such as *R. paludigena* SP9-15 (20.92 Mbp), ^15^ *R. paludigena* TL35-5 (20.98 Mbp), ^16^ *R. sampaioana* PL61-2, ^16^ *R. glutinis* ZHK (21.8 Mbp), ^59^ *R. glutinis* X-20 (21.85 Mbp), ^60^ *R. toruloides* VN1 (20.01 Mbp), ^13^ and *R. mucilaginosa* RIT389 (19.66 Mbp). ^47^ On the whole genome level, *R. mucilaginosa* HL26-1 has the highest average nucleotide identity of 99.75% to *R. mucilaginosa* strain JY1105. Similarly, the ANI of *R. paludigena* LL69-1 was greater at 99.58 than that of the nearest species, *R. paludigena* CM33. These high ANI values, typically ≥ 95%, underscore a strong correlation with other biological data, suggesting that the two yeasts likely belong to the same species. ^61^ Comparative genomic analysis revealed that the predicted number of protein-encoding genes of *R. mucilaginosa* HL26-1 is 5,711. In contrast, *R. paludigena* LL69-1 was predicted to contain approximately 6,782 coding genes. This result indicates that organisms of different species commonly possess varying quantities of protein-coding genes. In addition, critical genes involved in the terpenoid backbone and astaxanthin biosynthesis of these yeasts were analyzed based on functional genome annotation. All essential genes involved in terpenoid biosynthesis pathways were identified in the genomes of *R. paludigena* LL69-1 and strain HL26-1, except for the diphosphomevalonate decarboxylase gene ( *DPMDC*), which was not found in strain HL26-1. This error may have occurred during the genome sequencing procedure. An estimated 0.1–1% of processed bases will be sequenced incorrectly. ^62^

For the putative candidate astaxanthin synthesis-associated genes, the *CrtYB* (phytoene synthase/lycopene beta-cyclase), *CrtI* (phytoene desaturase), *CrtS* (beta-carotene 4-ketolase/3-hydroxylase), and *CrtR* (cytochrome P450) genes were identified and annotated in the genomes of both strains. The enzymes phytoene synthase/lycopene beta-cyclase and phytoene desaturase, encoded by *CrtYB* and *CrtI*, respectively, play important roles in the biosynthesis of beta-carotene, which is the precursor of astaxanthin synthesis. *Rhodotorula* species commonly produce β-carotene, torulene, and torularhodin at different ratios. ^63^ Additionally, we found that *CrtY* and *CrtB* were fused to strains HL26-1 and LL69-1 to form *CrtYB.* This corresponds to several *CrtYB*s found in various fungal species. ^64, 65^ These *CrtYB*s encode a protein with two functions: lycopene cyclase and phytoene synthase activities.

The *CrtS* gene encodes a specific astaxanthin synthase enzyme responsible for the ketolation and hydroxylation of β-carotene, facilitating the production of astaxanthin. ^66^ This enzymatic process is further augmented by the cytochrome P450 reductase enzyme *CrtR.* Originally identified in the pigmented yeast *Xanthophyllomyces dendrorhous,* ^67^ *CrtS* has since been identified in other pigmented yeasts, such as the genus *Rhodotorula.* ^15, 16, 18^ Our investigation specifically revealed the presence of all probable genes involved in astaxanthin biosynthesis in *R. mucilaginosa* HL26-1 and *R. paludigena* LL69-1. This finding provides compelling evidence for the capability of these strains to produce astaxanthin and offers valuable insights for future genetic engineering efforts aimed at enhancing astaxanthin synthesis. Furthermore, the yeasts HL26-1 and LL69-1 can utilize various carbon sources, including lignocellulosic sugars such as glucose, xylose, and arabinose (see Supplementary Table S2). Genome analysis also revealed that *R. mucilaginosa* HL26-1 and *R. paludigena* LL69-1 possess protein-coding genes involved in glucose, xylose, and arabinose utilization. Hence, these unconventional yeasts present great potential for producing astaxanthin, fatty acids, and other valuable products from low-cost sugars.

## Conclusion

In this study, various pigmented yeasts from the genus *Rhodotorula*, including *Rhodotorula mucilaginosa* and *Rhodotorula paludigena*, were isolated from flowers collected in Lampang Province. These yeasts demonstrate the ability to produce astaxanthin. Among these, *R. mucilaginosa* HL26-1 and *R. paludigena* LL69-1 exhibited the highest astaxanthin production among their respective species. Analysis of the draft genome sequences revealed the presence of several genes crucial for astaxanthin biosynthesis. These findings offer valuable insights for further advancements in the biotechnological and genomic applications of two promising astaxanthin-producing yeasts, *R. mucilaginosa* HL26-1 and *R. paludigena* LL69-1.

## Ethical approval

Not applicable.

## Data Availability

NCBI/GenBank: Whole-genome sequences of *R. mucilaginosa* HL26-1. GenBank accession numbers JAZBNE000000000; https://www.ncbi.nlm.nih.gov/nuccore/JAZBNE000000000.1, ^68^ Whole-genome sequences of *R. paludigena* LL69-1. GenBank accession numbers JAWJBI000000000; https://www.ncbi.nlm.nih.gov/nuccore/JAWJBI000000000.1. ^69^ Figshare: Supplementary information on characterization of red-pigmented yeasts and genes associated with astaxanthin synthesis, https://doi.org/10.6084/m9.figshare.28953866.v3. ^70^ This project contains the following underlying data: Supplymentary information_June4.pdf Data are available under the terms of the Creative Commons Attribution 4.0 International license (CC-BY 4.0).
