# Characterization of red pigmented yeasts and genes associated with astaxanthin synthesis in *Rhodotorula mucilaginosa* HL26-1 and *Rhodotorula paludigena* LL69-1

**DOI:** 10.12688/f1000research.164600.2

**Published:** 2026-01-02

**Authors:** Patcharaporn Hoondee, Nisachon Tedsree, Sukanya Phuengjayaem, Engkarat Kingkaew, Boonchoo Sritularak, Pornchai Rojsitthisak, Takuji Nakashima, Worathat Thitikornpong, Somboon Tanasupawat

**Affiliations:** 1 Division of Biology, Faculty of Science and Technology, Rajamangala University of Technology Krungthep, Sathon, Bangkok, 10120, Thailand; 2 Biodiversity and Sustainable Utilization Research Unit, Rajamangala University of Technology Krungthep, Sathon, Bangkok, 10120, Thailand; 3 Faculty of Science and Arts, Chanthaburi Campus, Burapha University, Tha Mai, Chanthaburi, 22170, Thailand; 4 Department of Microbiology, Faculty of Science, King Mongkut's University of Technology Thonburi, Thung Khru, Bangkok, 10140, Thailand; 5 Department of Biology, School of Science, King Mongkut's Institute of Technology Ladkrabang, Lat Krabang, Bangkok, 10520, Thailand; 6 Center of Excellence in Natural Products for Ageing and Chronic Diseases, Chulalongkorn University, Pathumwan, Bangkok, 10330, Thailand; 7 Department of Pharmacognosy and Pharmaceutical Botany, Faculty of Pharmaceutical Sciences, Chulalongkorn University, Pathumwan, Bangkok, 10330, Thailand; 8 Department of Food and Pharmaceutical Chemistry, Faculty of Pharmaceutical Sciences, Chulalongkorn University, Pathumwan, Bangkok, 10330, Thailand; 9 Department of Field Sciences, University of Human Environments, Mutsuyama, Ehime, 790-0825, Japan; 10 Research Organization for Nano and Life Innovation, Waseda University, Shinjuku, Tokyo, 162-0041, Japan; 11 Department of Biochemistry and Microbiology, Chulalongkorn University, Pathumwan, Bangkok, 10330, Thailand

**Keywords:** astaxanthin, pigmented yeast, Rhodotorula mucilaginosa, Rhodotorula paludigena, astaxanthin synthase

## Abstract

**Background:**

Astaxanthin, a red xanthophyll carotenoid, is a powerful antioxidant, anticancer, and glucose and lipid homeostasis regulator. Some pigmented yeasts belonging to the genus *Rhodotorula*, the well-known yeast for beta-carotene production, have been reported as natural astaxanthin producers. However, the lack of genomic data on astaxanthin-producing strains within these species hinders the identification of biosynthetic routes, molecular characterization of these pathways, and gene editing applications.

**Methods:**

This study explored the diversity and astaxanthin production capability of cultivable pigmented yeast in flower samples. The astaxanthin production ability was inspected by three consecutive methods, including thin-layer chromatography (TLC) for the preliminary step, followed by quantitative spectrophotometry and high-performance liquid chromatography (HPLC) for qualitative validation. The draft genome sequence and astaxanthin-producing genes of astaxanthin-producing yeasts were examined.

**Results:**

Twelve of 23 yeasts from floral samples exhibited natural pigmentation, with colors ranging from pinkish-orange to red, and exhibited the potential for astaxanthin synthesis. These yeasts were *Rhodotorula paludigena* (three strains) and *Rhodotorula mucilaginosa* (nine strains). Among *R. mucilaginosa* strains, HL26-1 had the greatest astaxanthin content (104.98 ± 0.13 μg/g DCW) and yield (0.9280 ± 0.0012 mg/L). Strain LL69-1 has the greatest astaxanthin content (251.78 ± 0.27 μg/g DCW) and yield (1.8632 ± 0.0023 mg/L) among *R. paludigena* strains. The 18.78 Mbp *R. mucilaginosa* HL26-1 genome includes 5,711 protein-coding genes. Conversely, the *R. paludigena* LL69-1 genome was 20.99 Mbp, encompassing 6,782 predicted genes. A comprehensive investigation of draft genome sequences of these two strains identified *CrtE*, *CrtYB*, *CrtI*, *CrtS*, and *CrtR* as potential astaxanthin transcription genes.

**Conclusion:**

Here, our results highlight the outstanding potential of two naturally pigmented yeasts, *R. mucilaginosa* HL26-1 and *R. paludigena* LL69-1, for astaxanthin production. Furthermore, our findings provide information on the whole genome and protein-encoded genes associated with astaxanthin production, which serve as valuable biological resources for various biotechnological applications.

## Introduction

Astaxanthin (3,3′-dihydroxy-β, β′-carotene-4,4′-dione) is a red pigment compound belonging to the xanthophyll carotenoid group and is widely recognized as a powerful antioxidant. The effective free radical–scavenging activity of astaxanthin contributes to its anticancer properties by reducing oxidative stress and singlet oxygen–mediated cellular damage. ^1^ Its antioxidant capacity has been reported to surpass that of vitamin E (α-tocopherol), β-carotene, canthaxanthin, and other natural carotenoids by up to 100-fold. ^2^ Beyond its antioxidant prowess, astaxanthin serves as a potent metabolic regulator of glucose and lipid homeostasis by increasing insulin sensitivity, augmenting glucose uptake, and modulating lipid turnover in the liver. In dermatology, it has garnered substantial attention for its ability to counteract oxidative stress, facilitate cellular rejuvenation, repair DNA damage, and protect against UV-induced photoaging and skin malignancies. ^3, 4^ Given these multifaceted biological activities, astaxanthin is extensively utilized across the pharmaceutical, nutraceutical, cosmetic, and animal feed industries. ^5^

Presently, astaxanthin is obtained either through chemical synthesis or natural extraction. ^6^ Despite its cost-effectiveness compared to natural sources, synthetic production often generates hazardous byproducts and poses environmental risks. ^7^ Consequently, the demand for natural sources such as shrimp, krill, fish, and microorganisms has surged. ^8^ Among microbes, the microalga *Haematococcus pluvialis* is the primary industrial source due to its high astaxanthin content. However, algal cultivation faces significant logistical and economic hurdles, including slow growth rate, susceptibility to contamination, and complex downstream processing. To overcome these limitations, recent research has shifted toward pigmented yeasts as alternative bio-factories. Compared to microalgae, yeasts offer several biotechnological advantages: they can be cultured in closed bioreactors, possess faster growth cycles, and can utilize various carbon sources, including lignocellulosic biomass and industrial by-products. ^6, 9– 11^

While *Phaffia rhodozyma* ( *Xanthophyllomyces dendrorhous*) has been the traditional yeast source for several decades, ^6, 12^ certain *Rhodotorula* species, such as *R. toruloides* ^13, 14^ *R. paludigena*, ^15, 16^ *R. mucilaginosa* ^17, 18^ and *Rhodotorula* sp., ^19^ have recently emerged as promising candidates for astaxanthin biosynthesis. Despite this potential, a critical research gap remains. The pathway of astaxanthin biosynthetic in *Phaffia rhodozyma* has been reported. ^20– 22^ The regulation starts with beta-carotene formation through mevalonate pathway. Acetyl-CoA was converted to isopentenyl-pyrophosphate (IPP), the precursor of all isoprenoids, which were further condensed to produce phytoene, the colorless carotenoid. Subsequently, phytoene was transformed into β-carotene, which was catalyzed by phytoene synthase/lycopene beta-cyclase and phytoene desaturase (encoded by *CrtYB* and *CrtI* genes, respectively). ^20– 22^ Finally, a single gene called *CrtS*, which could act as both a ketolase and a hydroxylase, was responsible for converting β-carotene into astaxanthin. ^21, 22^ However, the genomic information and metabolic pathways of astaxanthin-producing *Rhodotorula* species remain incompletely understood. Only a handful of studies have employed effective techniques, such as whole-genome sequencing and gene analysis, to elucidate genome-related astaxanthin production in *Rhodotorula* yeast. ^13, 15– 17^ As a result, the scarcity of comprehensive genomic data hampers the discovery of essential biosynthetic genes, molecular characterization, and the use of gene-editing technologies to increase production yields.

Flower tissues and nectar are recognized as important reservoirs for diverse yeast communities. Previous studies have reported that pigmented yeasts can function as growth-promoting microorganisms, ^23, 24^ have shown great potential as biosynthetic fungicides for the control of postharvest fruit decay, ^25, 26^ and may influence plant–pollinator dynamics through the modification of nectar chemistry. ^27^ Moreover, pigmented yeasts isolated from flowers are often stress-tolerant, with carotenoid pigments contributing to their stress resistance by providing photoprotection against UV-B radiation, thereby supporting yeast growth and enhancing survivorship in UV-exposed environments. ^28^ These yeasts are also capable of producing valuable carotenoid pigments, such as β-carotene and astaxanthin, making them promising candidates for a wide range of biotechnological applications. ^29^

This study aimed to isolate and characterize novel astaxanthin-producing yeast strains obtained from flowers. Furthermore, we investigated the genomic profiles of two chosen astaxanthin-producing yeast strains, namely, *Rhodotorula mucilaginosa* HL26-1 and *Rhodotorula paludigena* LL69-1. Through analysis of their whole-genome sequences and identification of genes associated with astaxanthin synthesis, our objective was to provide comprehensive insights into the repertoire of proteins encoded within their genomes. This endeavor holds promise for elucidating their functional capabilities and igniting excitement about their potential integration as valuable biological resources across a spectrum of biotechnological applications, from the food and feed industries to the pharmaceutical and cosmetic sectors.

## Methods

### Isolation and phenotypic characterization

We collected eleven flower samples from the residential areas in Lampang Province, Thailand, in April 2022. Samples were randomly cut and collected into a sterile plastic bag through a scissor flame sterilized. After sampling, the flowers were carefully transported as soon as possible to the laboratory at low temperature (4°C) to maintain integrity during transit. Flower identification was conducted on-site by interviewing the owner of the plant and was then identified by one of the co-authors (B.S.). Specimens have been deposited at the Department of Pharmacognosy and Pharmaceutical Botany, Faculty of Pharmaceutical Sciences, Chulalongkorn University, Thailand. Detailed information regarding the samples, specimen number, and their respective collection locations is provided in Table S1. The enrichment method has been used for yeast isolation. One gram of each sample was inoculated into 15 mL of yeast malt (YM) broth medium (10 g/L glucose, 5 g/L peptone, 3 g/L yeast extract, 3 g/L malt extract, pH 5.5). The inoculated media were incubated at 25°C for 3 days. Subsequently, the resulting cultures were streaked onto YM agar medium (pH 5.5) and incubated at 30°C for 5 days. Following incubation, pigmented yeast colonies displaying the desired pink to red hue were meticulously selected and purified on YM agar media using the streak plate method. Each isolate culture was carefully preserved on a YM agar slant at 4°C to facilitate further analysis and experimentation. Carbon assimilation of the strain was performed using the API ^®^ ID 32 C kit (BioMérieux, France) according to the manufacturer’s instructions. Reactions were visually examined at 72 hours, and then the results were interpreted to be positive or negative based on the presence or absence of turbidity in the carbohydrate wells.

### Molecular identification of yeast strains

*Genomic DNA extraction*

Genomic DNA was extracted from the pure yeast cultures using the glass bead extraction method ^30^ with some modification. Briefly, yeast cells were washed twice with sterilized distilled water and then lysed by vortexing with 0.3 g of 0.45-0.52 mm diameter acid washed glass beads in 200 μL of extraction buffer (comprising 2% (v/v) Triton X-100, 1% (w/v) SDS, 100 mM NaCl, 10 mM Tris-HCl (pH 8.0), and 1 mM EDTA (pH 8.0)) for 5 minutes. The resulting cell-free supernatant was transferred to a new tube and gently mixed with a 2X volume of phenol (Sigma–Aldrich ^®^, USA, Cat. No. 242322), chloroform (RCI Labscan ^®^, Ireland, Cat. No. AR 1027E), and isoamyl alcohol (Sigma–Aldrich ^®^, USA, Cat. No. 8.22255) at a ratio of 25:24:1. After centrifugation, the supernatant was transferred to a new tube containing 1 mL of absolute ethanol (RCI Labscan ^®^, Ireland, Cat. No. AR1069) and stored at -20°C for 20 minutes. The DNA pellet obtained after centrifugation was washed with 500 μL of 70% (v/v) ethanol, dried at 37°C, dissolved in 50 μL of TE buffer, and stored at -20°C until further use.

*Sequencing of the 26S rRNA gene (D1/D2 domain)*

The 26S rRNA gene within the D1/D2 domain of the large subunit (LSU D1/D2 domain) was amplified by PCR using the primers NL1 (5′-GCATATCAATAAGCGGAGGAAAAG-3′) and NL4 (5′-GGTCCGTGTTTCAAGACGG-3′). ^31^ PCR was conducted in a 20 μL volume comprising 2 μL of DNA template, 0.4 μL of each primer (10 pmol/μL), 10 μL of 2X Go Tag Green, and 7.2 μL of distilled water. The amplification process involved an initial denaturation step at 94°C for 3 minutes, followed by 36 cycles of denaturation at 94°C, annealing at 52°C, and extension at 72°C, each for 30 seconds, and a final extension step at 72°C for 5 minutes. Subsequently, the PCR products were purified using a gel/PCR DNA fragment extraction kit (GenepHlow™, Geneaid Biotech Ltd., Taiwan). Purified PCR products were sequenced bidirectionally using BT sequencing technology (Celemics Inc., Republic of Korea).

*Analysis of phylogenetic placement*

The LSU D1/D2 sequences were aligned with those of related species using MUSCLE, ^32^ and any gaps were removed. MEGA11 software ^33^ was used to construct a neighbor-joining (NJ) tree using Kimura’s two-parameter model. ^34^ The reliability of the branches was assessed using a bootstrap test with 1000 replicates. ^35^

### Yeast cultivation for astaxanthin production

A single loopful of yeast cultured on YM agar was transferred to YM broth (50 mL) in a 250 mL flask and then incubated at 30°C with agitation at 200 rpm for 24 hours. Subsequently, a 5 mL aliquot of this culture was inoculated into fresh YM broth (45 mL) in a 250 mL flask and incubated under the same conditions for 72 hours. Following incubation, the cells were harvested by centrifugation at 4°C and 6,500 × g, for 10 minutes, washed twice with distilled water, and then subjected to lyophilization for drying. Experiments were performed in triplicate. The resultant dry cell weight (DCW) was determined to quantify the cell biomass. Lyophilized cells were used for further analysis of astaxanthin.

### Astaxanthin analysis

*Qualitative analysis of astaxanthin production by thin layer chromatography*

The extraction and qualitative analysis of astaxanthin were performed using a slightly modified method described by Ushakumari and Ramanujan. ^36^ Lyophilized cells (0.01 g DCW) were suspended in 1 mL of acetone (RCI Labscan ^®^, Ireland, Cat. No. AR1003) and homogenized using a pestle at room temperature for 3 minutes. The supernatant was collected by centrifugation at 12,300 × g, for 10 minutes. Subsequently, an aliquot (20 μL) of the extract was applied onto a silica gel TLC plate using a capillary tube, alongside an astaxanthin standard. Analytical-grade astaxanthin with purity ≥ 98% was purchased from Dayang Chem Co. Ltd. (China) and utilized as a reference standard. Plate development was carried out using acetone:hexane (1:3, v/v) as the mobile phase. Acetone and hexane were purchased from RCI Labscan ^®^ (Ireland) with Cat. No. of AR1003 and AR1085, respectively. After development, colored bands were directly visualized under visible light and compared with the astaxanthin standard. The *R _f_* value was calculated to evaluate the migration of pigments on the TLC plate.

*Quantification of astaxanthin production*

The extraction and measurement of astaxanthin were conducted using previously described methods with slight modifications. According to Li and others ^37^ and Casella, ^38^ absorbance at 530 nm shows a strong linear correlation with astaxanthin content while minimizing interference from other carotenoids; therefore, this wavelength was used for astaxanthin quantification in this study. To extract the intracellular carotenoid content, 50 mg of lyophilized cells were suspended in 5 mL of dimethyl sulfoxide (DMSO; Sigma–Aldrich ^®^, USA, Cat. No. 34869) and ultrasonicated at 37 kHz and 50°C for 30 minutes using an ultrasonic bath (Elmasonic, E60H model, Germany). The extracts were centrifuged at 12,300 × g for 5 minutes. The extraction process was repeated until the supernatant became colorless. Astaxanthin concentration was determined using a Cary 60 UV–Vis spectrophotometer (Agilent) at 530 nm against a DMSO blank. A standard calibration curve was generated using astaxanthin concentration of 0, 0.25, 0.5, 1, 2, 4, 6, and 8 μg/mL prepared in DMSO. Astaxanthin concentrations were calculated based on the standard calibration curve, and the results are presented as the mean of triplicate measurements. Statistical analyses were performed using SPSS software for Windows (version 22). One-way analysis of variance (ANOVA) was applied to evaluate differences among individual factors, and Tukey’s multiple range test was used for post hoc pairwise comparisons at a significance level of α = 0.05. Astaxanthin production efficiency among the different strain was compared. The strains, which showed the highest astaxanthin production among their species, were selected for further study.

*Confirmative analysis of astaxanthin synthesis*

The astaxanthin was extracted and analyzed based on the described method. ^15^ In brief, lyophilized cells (0.05 g dry cell weight) were suspended in 5 mL of DMSO (Sigma–Aldrich ^®^, USA, Cat. No. 34869), mixed thoroughly, and sonicated at 37 kHz (Elmasonic E60H, Germany) at 55°C for 5 minutes. The mixture was centrifuged at 6,500 × g for 10 minutes and the resultanting supernatant was filtered through a 0.22 μm membrane filter prior to high performance liquid chromatography (HPLC) analysis. HPLC analysis was performed using a UHPLC Nexera X2 system (Shimadzu, Japan) equipped with an LC-30AD binary pump, SIL-30AC autosampler, CTO-20AC column oven and SPD-M30A detector, controlled by LabSolutions chromatography software. Seperation was achieved on a C18 column (GL Science InterSustain, 4.6 mm × 150 mm, 5 μm). The flow rate was set at 0.5 mL/minutes from 0.00 to 3.00 minutes and increased to 1.0 mL/minutes from 3.01 to 20.00 minutes. The detection wavelength was 480 nm, the column temperature was maintained at 30°C, and the injection volume was 5 μL. The mobile phase consisted of methanol (RCI Labscan ^®^, Ireland, Cat. No. LC1115)/acetonitrile (RCI Labscan ^®^, Ireland, Cat. No. LC1005)/ethyl acetate (RCI Labscan ^®^, Ireland, Cat. No. LC1070)/formic acid (Sigma–Aldrich ^®^, USA, Cat. No. 5.43804) (75.9:12:12:0.1, v/v) (solvent A) and methanol (solvent B).

### Whole-genome sequencing and analysis

The whole genome was sequenced using the paired-end (PE) 150 method on the Illumina HiSeq Xten/Novaseq/MGI2000 platform at Vishuo Biomedical Pte. Ltd., Beijing, China. The single-end reads were processed to eliminate adapters and low-quality bases using Fastp (v0.23.0). The resulting data were then assembled into contigs using Velvet de Novo assembler version 1.2.10. ^39, 40^ Subsequently, the contigs were assembled into scaffolds using SSPACE (version 3.0), ^41^ and the gaps were filled using GapFiller (versions 1–10). ^42^ Gene prediction was performed using Augustus version 3.3. ^43^ The coding genes were annotated using the National Center for Biotechnology Information (NCBI) NR database via BLAST. Subsequently, gene function prediction was carried out using the Kyoto Encyclopedia of Genes and Genomes (KEGG) ^44^ and KofamKOALA tools, employing default settings and considering all hits ( https://www.genome.jp/kegg/). ^45^ Proksee ( https://proksee.ca/) was used to generate the circular genome map, and OrthoVenn ( https://orthovenn3.bioinfotoolkits.net/ start/db) was used to create the Venn diagram. The Kostas Lab web-based tool ( http://enve-omics.ce.gatech.edu/) was used to analyze the average nucleotide identity (ANI) values. ^46^

## Results

### Isolation and identification of yeast strains

Twenty-three yeasts were isolated from the 11 flower samples collected (Table S1). Among them, 12 isolates exhibited pigmented colonies with the desired coloration ranging from pink to orange. All isolates were subjected to identification at the molecular operational taxonomic unit (MOTU) level by sequencing of the 26S rRNA gene (LSU D1/D2 domain), followed by species assignment via comparison with entries in the NCBI GenBank database using the BLASTn program. Consequently, all 12 pigment yeast strains were identified as basidiomycetous yeasts (Table S1), with two species belonging to the *Rhodotorula* genus: *Rhodotorula paludigena* (9 strains) and *Rhodotorula mucilaginosa* (3 strains). The remaining 11 strains were classified into four species of Basidiomycota— *Cryptococcus heveanensis* (1 strain), *Pseudozyma aphidis* (2 strains), *Pseudozyma hubeiensis* (1 strain), and *Pseudozyma siamensis* (1 strain)—and five species of Ascomycota— *Candida parapsilosis* (1 strain), *Metschnikowia koreensis* (1 strain), *Saccharomyces cerevisiae* (2 strains), *Wickerhamiella infanticola* (1 strain), and *Debaryomyces nepalensis* (1 strain).

### Qualitative analysis of astaxanthin production by thin layer chromatography

**Figure 1.**
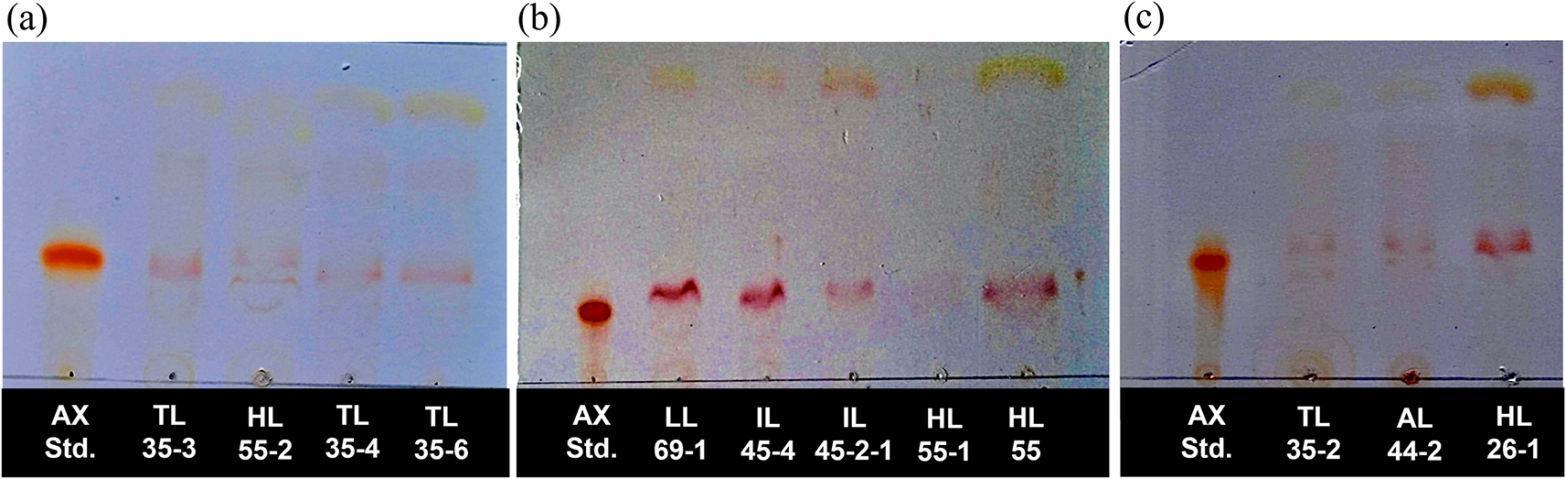
Thin layer chromatography (TLC) of an acetone extract of pigment isolated yeasts and astaxanthin standard on Silica gel G plate using a mixture of acetone and hexane (ratio of 1:3 (v/v)) as the mobile phase. The presence of astaxanthin band of *R. paludigena* strain TL35-3, HL55-2, TL35-4, and TL35-6 (a), strain LL69-1, IL45-1, IL45-2-1, HL55-1, HL55 (b), and *R. mucilaginosa* strain TL35-2, AL44-2, and HL26-1 (c).

A total of twelve pigmented strains were selected for qualitative analysis of astaxanthin production using thin-layer chromatography (TLC) technique. Astaxanthin, a red carotenoid, was directly visualized on the TLC plates without derivatization. Comparison with the astaxanthin standard revealed a corresponding band in all extracts. TLC analysis showed the presence of several pigment bands in the extracts. Astaxanthin was identified by comparing the *R _f_* value and coloration of the sample band with those of the astaxanthin standard analyzed under identical chromatographic conditions. The astaxanthin bands from all strains migrated within narrow *R _f_* range centered around 0.28, corresponding to the *R _f_* value of the astaxanthin standard. Minor variations in *R _f_* values were observed; however, all astaxanthin bands matched the standard, confirming the presence of astaxanthin in all tested yeast strains ( Figure 1). Based on these results, all yeast strains were selected for further quantitative analysis of astaxanthin content.

### Quantification of astaxanthin production

**Figure 2.**
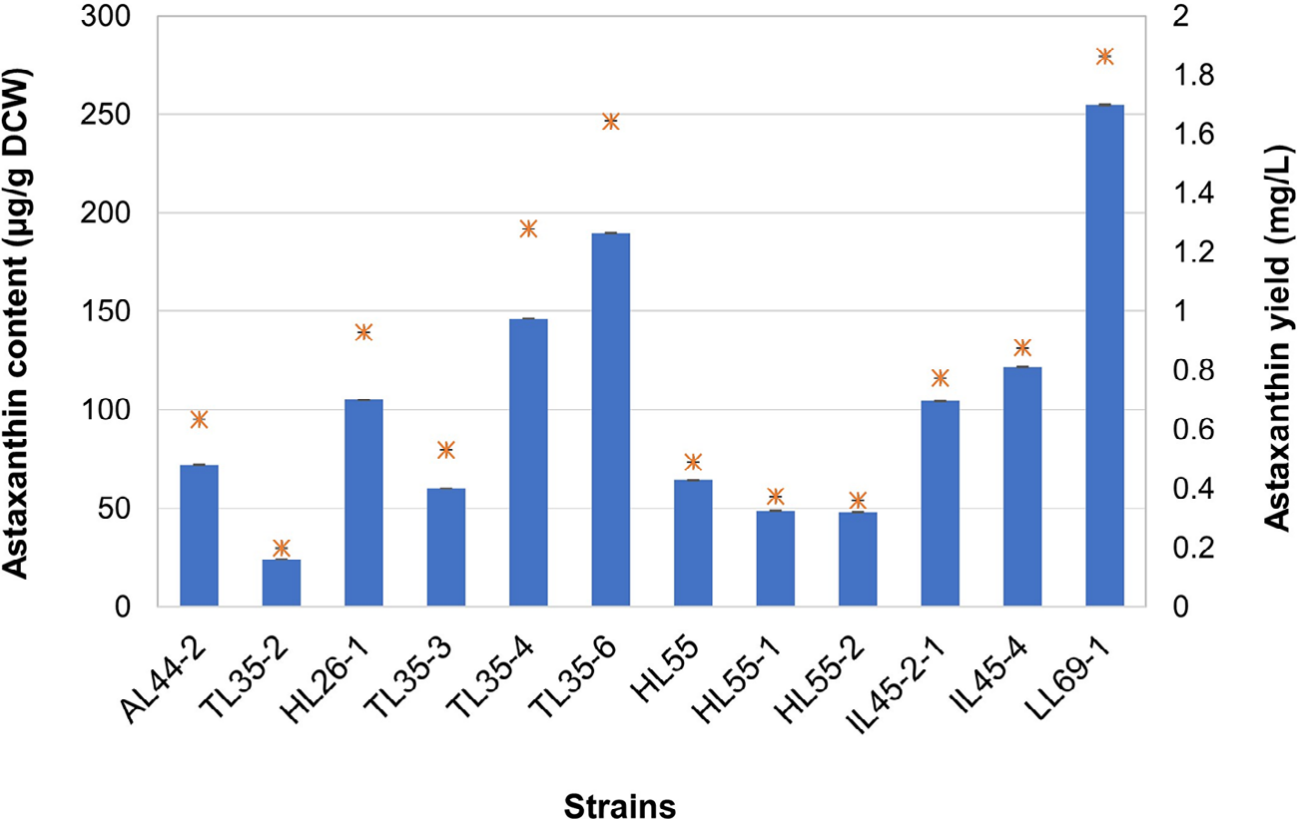
Quantitative analysis of astaxanthin production by 3 *R. mucilaginosa* strains (AL44-2, TL35-2, and HL26-1) and 9 *R. paludigena* strains (TL35-5, TL35-4, TL35-6, HL55, HL55-1, HL55-2, IL45-2-1, IL45-4, and LL69-1). The results are obtained from three replicate experiments and reported as the average value ± standard deviation (SD) for n = 3, with different letters indicating significant difference ( *p* < 0.05 one-way analysis of variance (ANOVA)).

Twelve yeast strains exhibiting positive results in TLC analysis for astaxanthin were subjected to quantitative analysis using spectrophotometry. Intracellular astaxanthin was extracted from powdery cells using the conventional DMSO extraction method ^37^ coupled with sound energy via an ultrasonic bath to enhance cell disruption efficiency. ^47^ In the *R. mucilaginosa* strains, the astaxanthin content ranged from 23.99 ± 0.22 to 104.98 ± 0.13 μg/g DCW, with yields ranging from 0.1991 ± 0.0021 to 0.9280 ± 0.0012 mg/L ( Figure 2). Moreover, several *R. paludigena* strains exhibited astaxanthin contents and yields ranging from 47.99 ± 0.11 to 251.78 ± 0.27 μg/g DCW and from 0.3589 ± 0.0009 to 1.8632 ± 0.0023 mg/L, respectively ( Figure 2). Strain LL69-1 demonstrated the highest astaxanthin production of *R. paludigena*, with an astaxanthin content of 251.78 ± 0.27 μg/g DCW and a yield of 1.8632 ± 0.0023 mg/L. Meanwhile, *R. mucilaginosa* strain HL26-1, the highest producer of its species, which yielded 104.98 ± 0.13 μg/g DCW and 0.9280 ± 0.0012 mg/L.

### Confirmative analysis of astaxanthin synthesis in selected astaxanthin-producing strains

The ability of two astaxanthin-producing yeasts, *R. mucilaginosa* HL26-1 and *R. paludigena* LL69-1, to synthesize astaxanthin was confirmed by high-performance liquid chromatography (HPLC). The HPLC chromatogram of both strains exhibited peaks corresponding to astaxanthin when compared with a 10 ppm astaxanthin standard. The retention times of the predominant peaks were observed at 2.061 minutes for strain HL26-1 and 2.211 minutes for strain LL69-1. Astaxanthin was identified by comparing the retention times of the sample peaks with that of the authentic astaxanthin standard. In addition, spiking the sample extracts with the astaxanthin standard resulted in a proportional increase in the corresponding peak area without the appearance of additional peaks, confirming the identity of astaxanthin. Representative HPLC chromatograms of the standard, samples, and spiked samples are shown in Supplementary Figure S1. These results demonstrate that both HL26-1 and LL69-1 are capable of astaxanthin production.

### General features of the *R. paludigena* LL69-1 and *R. mucilaginosa* HL26-1 genomes

**Figure 3.**
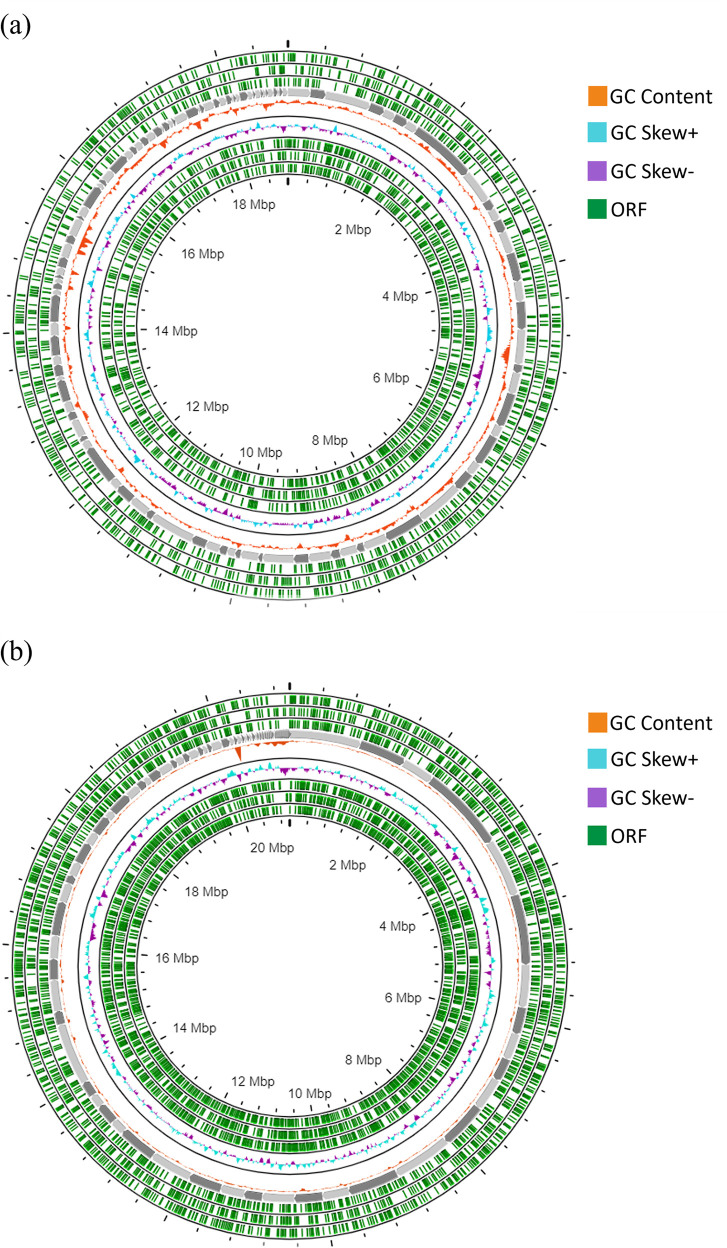
Circular genomic map of *R. mucilaginosa* HL26-1 and *R. paludigena* LL69-1 displaying the following information: GC content in orange, GC skew (+) in blue, GC skew (-) in purple, and open reading frames (ORFs) in green.

The genome assembly statistics for the two astaxanthin-producing yeasts, *R. mucilaginosa* HL26-1 and *R. paludigena* LL69-1, are presented in Table 1. The assembly of *R. mucilaginosa* HL26-1 resulted in 107 scaffolds, with the giant scaffold spanning 853,611 base pairs (bp). The N50 length of the scaffolds was 193,129 bp, with a GC content of 60.12% and a genome size of 18.78 Mbp. Conversely, *R. paludigena* LL69-1 had a genome size of 20.99 Mbp, with a GC content of 63.82%. Its assembly comprised 107 scaffolds with an N50 value of 476521 bp and an L50 value of 24 scaffolds. The largest scaffold in LL69-1 was 1,230,191 bp, whereas the shortest was 524 bp. Circular genomics of the HL26-1 and LL69-1 genomes, illustrating the open reading frame (ORF) positions, GC content, and GC skew of each strain, are depicted in Figure 3. The whole-genome sequences of *R. mucilaginosa* HL26-1 and *R. paludigena* LL69-1 were deposited in the NCBI/GenBank database ( http://www.ncbi.nlm.nih.gov), associated with the BioProject identities PRJNA1025132 and PRJNA1025134, BioSample numbers SAMN37714267 and SAMN37714269, and GenBank accession numbers JAZBNE000000000 and JAWJBI000000000, respectively.

**Table 1.** Assembly statistics of the *R. paludigena* HL26-1 and *R. mucilaginosa* LL69-1 genomes.

Features	Strains	
	*R. mucilaginosa* HL26-1	*R. paludigena* LL69-1
Genome assembly size (bp)	18,775,076	20,987,037
Max length (bp)	853,611	1,230,191
Number of scaffolds	107	154
N50 (bp)	193,129	476,521
GC (%)	60.12	63.82
Predicted protein-coding gene	5,711	6,782

### Phylogenetic and average nucleotide identity (ANI) analysis of strains

Figure S2 depicts a phylogenetic tree using the D1/D2 domains of the large subunit sequences obtained in this study and those available in the NCBI nucleotide database. This analysis confirmed the phylogenetic relationship between these pigmented yeasts and other related species. *R. mucilaginosa* HL26-1 and *R. paludigena* LL69-1 were clustered within the same clade as their closest-type strains, *R. mucilaginosa* CBS316 ^T^ and *R. paludigena* CBS6566 ^T^, respectively, with 100% bootstrap support.

Moreover, we conducted an average nucleotide identity (ANI) analysis, a crucial step in assessing the phylogenomic relationships between the two astaxanthin-producing yeasts and other *Rhodotorula* species. The pairwise ANI values of the whole genomes of *R. mucilaginosa* HL26-1, *R. paludigena* LL69-1, and the other *Rhodotorula* species varied (Supplementary Figure S3).

### Functional genome annotation and comparative analysis of putative gene families associated with astaxanthin biosynthesis

**Figure 4.**
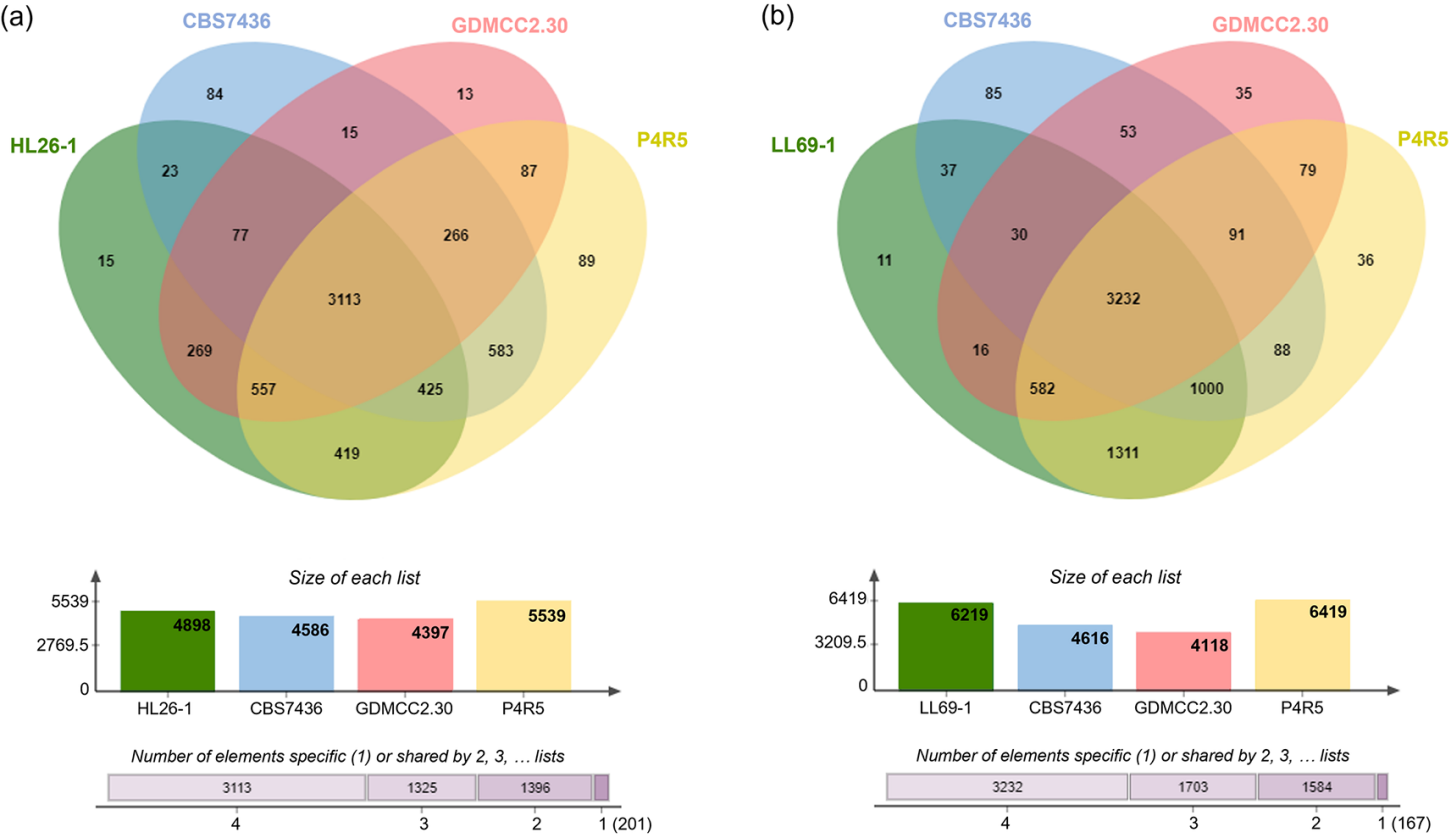
Venn diagram displaying the presence of conserved and specific proteins among *Rhodotorula mucilaginosa* HL26-1 (a) or *R. paludigena* LL69-1 (b) and *R. kratochvilovae* CBS 7436, *R. mucilaginosa* GDMCC2.30, and *R. paludigena* P4R5.

According to annotation results from the National Center for Biotechnology Information (NCBI) database, the draft genome sequence of *R. mucilaginosa* HL26-1 comprises 5,711 protein-encoding genes, while *R. paludigena* LL69-1 was predicted to contain approximately 6,782 coding genes. The Venn diagram in Figure 4 illustrates the genetic variations and distinct characteristics that differentiate the genomes of HL26-1 and LL69-1 from those of closely related species. The protein-coding sequences of HL26-1 and LL69-1, along with those of three other *Rhodotorula* species ( *R. kratochvilovae* CBS 7436, *R. mucilaginosa* GDMCC2.30, and *R. paludigena* P4R5), were compared to examine the similarity of their protein sequences. This comparison revealed that *R. mucilaginosa* HL26-1, *R. kratochvilovae* CBS 7436 *, R. mucilaginosa* GDMCC2.30, and *R. paludigena* P4R5 possess 15, 84, 13, and 89 proteins, respectively, which are exclusive to their respective species or strains. Additionally, 11, 85, 35, and 36 protein families were identified as being species- or strain-specific for *R. paludigena* LL69-1, *R. kratochvilovae* CBS 7436 ^T^ *, R. mucilaginosa* GDMCC2.30, and *R. paludigena* P4R, respectively.

KEGG metabolic pathway analysis categorized a total of 2,795 (52.1%) protein-coding genes of HL26-1 into four major groups: metabolism (951 genes), genetic information processing (682 genes), cellular activities (225 genes), and environmental information processing (137 genes). Within the LL69-1 dataset, 3,303 protein-coding genes (48.7%) were subjected to KEGG analysis and classified into four major categories: metabolism (1,064 genes), genetic information processing (734 genes), cellular activities (245 genes), and environmental information processing (168 genes).

The putative gene families associated with astaxanthin biosynthesis in strains HL26-1 and LL69-1 were determined ( Table 2). The pathway of astaxanthin synthesis was divided into two main sections: one that creates the basic building blocks called terpenoids, which are important natural substances, and another that specifically produces astaxanthin.

**Table 2.** Putative gene families associated with astaxanthin biosynthesis in *R. mucilaginosa* HL26-1 and *R. paludigena* LL69-1.

Pathways	Putative genes	Enzyme product (KEGG orthologs number, EC number)	LL69-1		HL26-1	
			Scaffold ID	E-value	Scaffold ID	E-value
Terpenoid backbone biosynthesis	*ACAT*	Acetyl-CoA acetyltransferase (K00626, EC:2.3.1.9)	Scaffold13.g2648	2.1×10 ^−168^	Scaffold38.g4472	5.1×10 ^−172^
	*HMGCS*	Hydroxymethylglutaryl-CoA synthase (K01641, EC:2.3.3.10)	Scaffold18.g3608	7.1×10 ^−197^	Scaffold33.g3151	6.4×10 ^−194^
	*HMGCR*	Hydroxymethylglutaryl-CoA reductase (K00021, EC:1.1.1.34)	Scaffole80.g6723	3.0×10 ^−296^	Scaffold61.g2990	9.1×10 ^−298^
	*MVD*	Mevalonate kinase (K00869, EC:2.7.1.36)	Scaffold13.g2759	2.5×10 ^−108^	Scaffold39.g1654	7.3×10 ^−103^
	*PMK*	Phosphomevalonate kinase (K00938, EC:2.7.4.2)	Scaffold13.g2880	7.2×10 ^−122^	Scaffold38.g4446	1.0×10 ^−121^
	*DPMDC*	Diphosphomevalonate decarboxylase (K01597, EC:4.1.1.33)	Scaffold58.g6593	2.1×10 ^−150^	Undetectable	-
	*IDI*	Isopentenyl-diphosphate delta isomerase (K01823, EC:5.3.3.2)	Scaffold13.g2706	1.5×10 ^−67^	Scaffold39.g1606	8.8×10 ^−68^
	*GGDPS*	Geranylgeranyl diphosphate synthase (K00804, EC:2.5.1.1 2.5.1.10 2.5.1.29)	Scaffold4.g827	1.0×10 ^−149^	Scaffold103.g709	5.6×10 ^−148^
	*FDPS*	Farnesyl diphosphate synthase (K00787, EC:2.5.1.1 2.5.1.10)	Scaffold14.g3047	1.1×10 ^−154^	Scaffold77.g4986	1.3×10 ^−155^
	*CrtE*	Geranylgeranyl pyrophosphate synthase (K05355, EC:2.5.1.82 2.5.1.83)	Scaffold1.g227	2.2×10 ^−168^	Scaffold17.g303	7.0×10 ^−171^
Astaxanthin biosynthesis	*CrtYB*	Phytoene synthase/lycopene beta-cyclase (K17841, EC:2.5.1.32 5.5.1.19)	Scaffold3.g680	7.9×10 ^−183^	Scaffold38.g4383	8.5×10 ^−170^
	*CrtI*	Phytoene desaturase (K15745, EC:1.3.99.30)	Scaffold3.g682	2.8×10 ^−234^	Scaffold38.g4386	9.8×10 ^−237^
	*CrtS*	Beta-carotene 4-ketolase/3-hydroxylase (K23037, EC:1.14.99.63 1.14.15.24 1.14.99.-)	Scaffold27.g4592	3.5×10 ^−81^	Scaffold49.g3491	6.2×10 ^−73^
	*CrtR*	Cytochrome P450 (K14338, EC:1.14.14.1 1.6.2.4)	Scaffold6.g1523	8.4×10 ^−68^	Scaffold42.g2465	2.9×10 ^−70^

The key genes associated with terpenoid biosynthesis discovered in the genomes of both strains were *ACAT* (acetyl-CoA acetyltransferase), *HMGCS* (hydroxymethylglutaryl-CoA synthase), *HMGCR* (hydroxymethylglutaryl-CoA reductase), *MVD* (mevalonate kinase), *PMK* (phosphomevalonate kinase), *IDI* (isopentenyl-diphosphate delta isomerase), *GGDPS* (geranylgeranyl diphosphate synthase), *FDPS* (farnesyl diphosphate synthase), and *CrtE* (geranylgeranyl pyrophosphate synthase). The diphosphomevalonate decarboxylase ( *DPMDC*) gene has only been found in strain LL69-1. The multiple gene involved in astaxanthin biosynthesis were annotated in the genomes of both strains, including the *CrtYB* (phytoene synthase/lycopene beta-cyclase), *CrtI* (phytoene desaturase), *CrtS* (beta-carotene 4-ketolase/3-hydroxylase), and *CrtR* (cytochrome P450) genes.

## Discussion

The pigmented yeast in the genus *Rhodotorula* is well established in yeast biotechnology applications and holds promise in numerous industrial sectors, including biofuels, carotenoids, enzymes, bioremediation, cosmetics, and biocontrol agents. ^48– 51^ Recently, some *Rhodotorula* species have exhibited unique abilities to naturally generate astaxanthin, a red pigment with excellent antioxidant activity. These species include *R. paludigena*, ^15, 16^ *R. sampaioana*, ^16^ *Rhodotorula mucilaginosa*, ^18^ and *R. toruloides*. ^13, 14^ Several *Rhodotorula* species have been isolated in this study. The most prevalent was *R. paludigena* (75%), which was found in the African marigold, pink West Indian jasmine, yellow hibiscus, and lantana flower samples. The remaining three *R. mucilaginosa* strains were isolated from flower samples of yellow West Indian jasmine, marigold, and hydrangea. The *Rhodotorula* genus is widespread and commonly found in diverse natural habitats, including nectar and floral tissue. ^28, 52, 53^ Earlier studies reported several flower-isolated pigmented yeasts had the ability to produce carotenoid pigment. *R. paludigena* SP9-15 isolated from Zinnia flowers ( *Zinnia violacea* Cav.), *R. paludigena* TL35-5 that inhabited Marigold flowers ( *Tagetes erecta*), *Rhodotorula* sp. CP72-2 isolated from *Calotropis gigantea* flowers, *R. sampaioana* PL61-2 isolated from white desert rose flowers ( *Plumeria obtusa*), and *Rhodosporidiobolus ruineniae* SP3-3/4 Red Cat’s Tail ( *Acalypha hispida* Burm.f.) flower exhibited astaxanthin production. ^15, 16, 19^ Moreover, the β-carotene-producing strain, *R. graminis* TY-99, isolated from flowers, was efficient for SCP production from waste milk. ^54^

Three investigation techniques were implemented to assess the capacity to generate astaxanthin. In the prescreening step, the TLC technique is considered reliable and accurate. Additionally, several studies have widely used TLC to confirm the presence of astaxanthin. ^55– 57^ For the specific quantification of astaxanthin, spectrophotometry has been used. ^37, 38, 58^ Meanwhile, high-performance liquid chromatography (HPLC) has been used as a qualitative confirmative analysis technique for astaxanthin synthesis. All twelve *Rhodotorula* strains identified in this study had the capability to synthesize astaxanthin, a distinctive and uncommon trait among this genus. Nine strains of *R. paludigena* exhibited astaxanthin contents ranging from 47.99 ± 0.11 to 251.78 ± 0.27 μg/g DCW and yields ranging from 0.3589 ± 0.0009 to 1.8632 ± 0.0023 mg/L, respectively. Meanwhile, the astaxanthin content was between 23.99 ± 0.22 and 104.98 ± 0.13 μg/g DCW, and the yields were between 0.1991 ± 0.0021 and 0.9280 ± 0.0012 mg/L for three *R. mucilaginosa* strains. Notably *, R. paludigena* LL69-1 exhibited the highest astaxanthin production, with an astaxanthin content and yield of 251.78 ± 0.27 μg/g DCW and 1.8632 ± 0.0023 mg/L, respectively, surpassing strain HL26-1, the highest producer of the *R. mucilaginosa* species, which yielded 104.98 ± 0.13 μg/g DCW of astaxanthin and 0.9280 ± 0.0012 mg/L of yield. These findings suggest that the ability to produce astaxanthin is species independent, as varying results have been obtained from diverse strains of the same species.

A comparison of the astaxanthin yield (mg/L) among the strains investigated in this study revealed that *R. paludigena* LL69-1 exhibited the highest astaxanthin production (1.86 mg/L) under the tested, non-optimized culture conditions. This yield was higher than those reported of other wild-type yeast strains cultivated under comparable or non-optimized conditions, such as *Phaffia rhodozyma* (0.2-0.4 mg/L) ^59^ and *R. toruloides* (0.93 mg/L), ^60^ indicating the promising potential of strain LL69-1 as a natural astaxanthin-producing yeast. However, the astaxanthin production of LL69-1 remains lower than that of many natural or genetically modified strains cultivated under optimized conditions. ^61^^,^^62^ For example, *Phaffia rhodozyma* 7B12 (originated from *P. rhodozyma* Past-1) produced 7.71 mg/L astaxanthin when cultivated in an optimal nitrogen medium consisting of 0.28 g/L (NH _4_) _2_SO _4_, 0.49 g/L KNO _3_, and 1.19 g/L beef extract. ^61^ The wild strain *Xanthophyllomyces dendrorhous* TISTR 5730 grown in mustard waste precipitated hydrolysate (MPH) under optimal conditions gave the astaxanthin yield of 25.8 mg/L, ^62^ while the *X. dendrorhous* strain DW6 produced 374.3 mg/L of astaxanthin using cane molasses in a two-stage pH fermentation system. ^63^ Similarly, higher astaxanthin yields have been reported for other *Rhodotorula* strains under optimized conditions, including *Rhodotorula* sp. CP72-2 (4.13 mg/L) ^19^ and *Rhodotorula paludigena* SP9-15 (6.67 mg/L). ^15^ These comparisons highlight that, although LL69-1 demonstrated the highest astaxanthin yield among the strains examined in this study under the tested conditions, further optimization of culture parameters is essential to enhance its production capacity. Moreover, additional genetic and metabolic information related to astaxanthin biosynthesis in *R. paludigena* and *R. mucilaginosa* is needed to support future strain improvement and process optimization.

The genomic information of two recently obtained astaxanthin-producing yeasts, *R. paludigena* LL69-1 and *R. mucilaginosa* HL26-1, is presented here. The genome sizes of *R. paludigena* LL69-1 and *R. mucilaginosa* HL26-1 were 18.78 Mbp with a GC content of 60.12% and 20.99 Mbp with a GC content of 63.82%, respectively. These findings align with the genome sizes of other pigmented yeast species such as *R. paludigena* SP9-15 (20.92 Mbp), ^15^ *R. paludigena* TL35-5 (20.98 Mbp), ^16^ *R. sampaioana* PL61-2, ^16^ *R. glutinis* ZHK (21.8 Mbp), ^64^ *R. glutinis* X-20 (21.85 Mbp), ^65^ *R. toruloides* VN1 (20.01 Mbp), ^13^ and *R. mucilaginosa* RIT389 (19.66 Mbp). ^66^ On the whole genome level, *R. mucilaginosa* HL26-1 has the highest average nucleotide identity of 99.75% to *R. mucilaginosa* strain JY1105. Similarly, the ANI of *R. paludigena* LL69-1 was greater at 99.58 than that of the nearest species, *R. paludigena* CM33. These high ANI values, typically ≥ 95%, underscore a strong correlation with other biological data, suggesting that the two yeasts likely belong to the same species. ^67^ Comparative genomic analysis revealed that the predicted number of protein-encoding genes of *R. mucilaginosa* HL26-1 is 5,711. In contrast, *R. paludigena* LL69-1 was predicted to contain approximately 6,782 coding genes. This result indicates that organisms of different species commonly possess varying quantities of protein-coding genes. In addition, critical genes involved in the terpenoid backbone and astaxanthin biosynthesis of these yeasts were analyzed based on functional genome annotation. All essential genes involved in terpenoid biosynthesis pathways were identified in the genomes of *R. paludigena* LL69-1 and strain HL26-1, except for the diphosphomevalonate decarboxylase gene ( *DPMDC*), which was not found in strain HL26-1. This error may have occurred during the genome sequencing procedure. An estimated 0.1–1% of processed bases will be sequenced incorrectly. ^68^

For the putative candidate astaxanthin synthesis-associated genes, the *CrtYB* (phytoene synthase/lycopene beta-cyclase), *CrtI* (phytoene desaturase), *CrtS* (beta-carotene 4-ketolase/3-hydroxylase), and *CrtR* (cytochrome P450) genes were identified and annotated in the genomes of both strains. The enzymes phytoene synthase/lycopene beta-cyclase and phytoene desaturase, encoded by *CrtYB* and *CrtI*, respectively, play important roles in the biosynthesis of beta-carotene, which is the precursor of astaxanthin synthesis. *Rhodotorula* species commonly produce β-carotene, torulene, and torularhodin at different ratios. ^69^ Additionally, we found that *CrtY* and *CrtB* were fused to strains HL26-1 and LL69-1 to form *CrtYB.* This corresponds to several *CrtYB*s found in various fungal species. ^70, 71^ These *CrtYB*s encode a protein with two functions: lycopene cyclase and phytoene synthase activities.

The *CrtS* gene encodes a specific astaxanthin synthase enzyme responsible for the ketolation and hydroxylation of β-carotene, facilitating the production of astaxanthin. ^72^ This enzymatic process is further augmented by the cytochrome P450 reductase enzyme *CrtR.* Originally identified in the pigmented yeast *Xanthophyllomyces dendrorhous,* ^73^ *CrtS* has since been identified in other pigmented yeasts, such as the genus *Rhodotorula.* ^15, 16, 19^ Our investigation specifically revealed the presence of all probable genes involved in astaxanthin biosynthesis in *R. mucilaginosa* HL26-1 and *R. paludigena* LL69-1. This finding provides compelling evidence for the capability of these strains to produce astaxanthin and offers valuable insights for future genetic engineering efforts aimed at enhancing astaxanthin synthesis. Furthermore, the yeasts HL26-1 and LL69-1 can utilize various carbon sources, including lignocellulosic sugars such as glucose, xylose, and arabinose (see Supplementary Table S2). Genome analysis also revealed that *R. mucilaginosa* HL26-1 and *R. paludigena* LL69-1 possess protein-coding genes involved in glucose, xylose, and arabinose utilization. Hence, these unconventional yeasts present great potential for producing astaxanthin, fatty acids, and other valuable products from low-cost sugars.

## Conclusion

In this study, various pigmented yeasts from the genus *Rhodotorula*, including *Rhodotorula mucilaginosa* and *Rhodotorula paludigena*, were isolated from flowers collected in Lampang Province. These yeasts demonstrate the ability to produce astaxanthin. Among these, *R. mucilaginosa* HL26-1 and *R. paludigena* LL69-1 exhibited the highest astaxanthin production among their respective species. Analysis of the draft genome sequences revealed the presence of several genes crucial for astaxanthin biosynthesis. These findings offer valuable insights for further advancements in the biotechnological and genomic applications of two promising astaxanthin-producing yeasts, *R. mucilaginosa* HL26-1 and *R. paludigena* LL69-1.

## Ethical approval

Not applicable.

## Data Availability

NCBI/GenBank: Whole-genome sequences of *R. mucilaginosa* HL26-1. GenBank accession numbers JAZBNE000000000; https://www.ncbi.nlm.nih.gov/nuccore/JAZBNE000000000.1, ^74^ Whole-genome sequences of *R. paludigena* LL69-1. GenBank accession numbers JAWJBI000000000; https://www.ncbi.nlm.nih.gov/nuccore/JAWJBI000000000.1. ^75^ Figshare: Supplementary information on characterization of red-pigmented yeasts and genes associated with astaxanthin synthesis, https://doi.org/10.6084/m9.figshare.28953866.v3. ^76^ This project contains the following underlying data: Supplymentary information_June4.pdf Data are available under the terms of the Creative Commons Attribution 4.0 International license (CC-BY 4.0).
