# Supplementary material for: Characterization of red pigmented yeasts and genes associated with astaxanthin synthesis in Rhodotorula mucilaginosa HL26-1 and Rhodotorula paludigena LL69-1
Source: F1000Res. 2026 Jan 2;14:717. Originally published 2025 Jul 21. [Version 2] doi: 10.12688/f1000research.164600.2 (PMC12817125; doi:10.12688/f1000research.164600.2)
Supplement: Peer review history

# Reviewer report: Reviewer response for version 2

**DOI:** 10.5256/f1000research.194508.r446928

**Published:** 2026-01-08

**Authors:** Kokati Venkata Bhaskara Rao

**Affiliations:** 1 Vellore Institute of Technology, Vellore, India

I have carefully evaluated the revised manuscript and find that the authors have satisfactorily addressed all the comments. The revisions have significantly improved the clarity, methodological rigor, and consistency of the manuscript. The introduction and Materials and Methods sections are now well structured and comprehensive, and issues related to astaxanthin quantification, genome statistics, and formatting have been appropriately resolved.

 I am satisfied with the revisions and recommend the manuscript for indexing.

Is the work clearly and accurately presented and does it cite the current literature?

Yes

If applicable, is the statistical analysis and its interpretation appropriate?

Partly

Are all the source data underlying the results available to ensure full reproducibility?

Yes

Is the study design appropriate and is the work technically sound?

Yes

Are the conclusions drawn adequately supported by the results?

Yes

Are sufficient details of methods and analysis provided to allow replication by others?

Yes

Reviewer Expertise:

Microbiology, drug discovery, pharmacology, Biotechnology, Biodegradation

I confirm that I have read this submission and believe that I have an appropriate level of expertise to confirm that it is of an acceptable scientific standard.

# Reviewer report: Reviewer response for version 1

**DOI:** 10.5256/f1000research.181137.r436643

**Published:** 2025-12-29

**Authors:** Kokati Venkata Bhaskara Rao

**Affiliations:** 1 Vellore Institute of Technology, Vellore, India

This study presents a well-executed investigation into the astaxanthin production capability of novel *Rhodotorula* strains isolated from flowers and provides valuable draft whole-genome sequences and gene annotations for the two highest-producing strains. The multi-step approach for astaxanthin confirmation (TLC, spectrophotometry, and HPLC) is scientifically sound and robust. The new genomic data and the finding that these yeasts can utilize lignocellulosic sugars are a significant contribution to the field of industrial microbiology and biotechnological astaxanthin production.

 1.Is the work clearly and accurately presented and does it cite the current literature? Yes

 2.Is the study design appropriate and is the work technically sound? Yes

 3. Are sufficient details of methods and analysis provided to allow replication by others? Yes

 4. If applicable, is the statistical analysis and its interpretation appropriate? Partly needs revision on adding statistical method used and p values has to be given to ensure reliability of the data

 5. Are all the source data underlying the results available to ensure full reproducibility? Yes

 6.Are the conclusions drawn adequately supported by the results? yes   

**Comments: Major Revision Required**

 The work is relevant for biotechnology, provides useful genomic insights, and adds value to the limited genomic data available for Rhodotorula paludigena and R. mucilaginosa. The manuscript is generally well-structured,however, several methodological and interpretative issues must be addressed before acceptance.

**1.Inconsistent Genome Assembly Statistics (Critical Error):** There is a serious and confusing inconsistency in the reporting of the draft genome sizes for the two selected strains. This must be corrected to maintain the authenticity of the data. Abstract states that the *R. mucilaginosa* HL26-1 genome is 18.78 Mbp, and the *R. paludigena* LL69-1 genome is 20.99 Mbp. The results section states that *R. mucilaginosa* HL26-1 assembly resulted in a genome size of 20.99 Mbp, and *R. paludigena* LL69-1 had a genome size of 18.78 Mbp. similar controversy in table 1.

**2.Astaxanthin Yield Comparison:** The discussion rightly notes that the high astaxanthin yield of *R. paludigena* LL69-1 (1.186mg/L) is superior to that of "other microorganisms". However, the paper later cites *Rhodotorula sp.* CP72-2 (4.13mg/L) and *R. paludigena* SP9-15 (6.67mg/L) ephrase the statement on superiority to be more precise, perhaps by comparing it only to unoptimized strains or stating it is the highest yield in this study under the tested conditions, to avoid contradicting the later discussion. The final conclusion that optimizing conditions is necessary is appropriate.

**3.Clarity on Strain Selection:** In the Abstract, it is stated that HL26-1 had the greatest content and yield among *R. mucilaginosa* strains, and LL69-1 had the greatest content and yield among *R. paludigena* strains. While clear, you may consider clarifying in the Methods why only two strains ( *R. mucilaginosa* HL26-1 and *R. paludigena* LL69-1) were chosen for the full whole-genome sequencing and analysis (presumably because they were the best producers of their respective species).

**4.Distinction between Carotenoids and Astaxanthin:** The paper must consistently and clearly differentiate between Total Carotenoids and Astaxanthin. In the Results and Discussion, ensure that when values are presented, it is clear whether they refer to the total pool of pigments (carotenes, torulene, etc.) or specifically to the astaxanthin fraction, which was isolated and quantified by HPLC. The difference is vital for biotechnological assessment.

**5.HPLC**: In the methods section, add chromatogram specifications, wavelength, flow rate, and injection volume.

**6.TLC: **TLC Rf value identical for all strains is suspicious—discuss variability **.**

**7.Astaxanthin standard: **Specify manufacturer purity in Methods **.**

**8.Statistical Analysis Missing: **Spectrophotometric data show mean ± SD, but no statistical tests are described. Mention statistical methods used to compare strains and add p-values where necessary.

Is the work clearly and accurately presented and does it cite the current literature?

Yes

If applicable, is the statistical analysis and its interpretation appropriate?

Partly

Are all the source data underlying the results available to ensure full reproducibility?

Yes

Is the study design appropriate and is the work technically sound?

Yes

Are the conclusions drawn adequately supported by the results?

Yes

Are sufficient details of methods and analysis provided to allow replication by others?

Yes

Reviewer Expertise:

Microbiology, drug discovery, pharmacology, Biotechnology, Biodegradation

I confirm that I have read this submission and believe that I have an appropriate level of expertise to confirm that it is of an acceptable scientific standard, however I have significant reservations, as outlined above.

# Response

**Published:** 2025-12-30

**Authors:** Somboon Tanasupawat

**Affiliations:** Biochemistry and Microbiology, Chulalongkorn University, Bangkok, Thailand

Dear reviewer,

 We sincerely thank the reviewer for these valuable comments. The suggestions provided have been carefully considered and will be incorporated into the revised version of the manuscript. A detailed point-by-point response addressing each of the reviewer’s comments is provided below.

 1.Inconsistent Genome Assembly Statistics (Critical Error): There is a serious and confusing inconsistency in the reporting of the draft genome sizes for the two selected strains. This must be corrected to maintain the authenticity of the data. The abstract states that the *R. mucilaginosa* HL26-1 genome is 18.78 Mbp, and the *R. paludigena* LL69-1 genome is 20.99 Mbp. The results section states that *R. mucilaginosa* HL26-1 assembly resulted in a genome size of 20.99 Mbp, and *R. paludigena* LL69-1 had a genome size of 18.78 Mbp. similar controversy in table 1.

 Answer: Thank you very much for pointing out this critical inconsistency. We sincerely apologize for this major error in reporting the genome assembly data, which occurred during manuscript preparation. We have now carefully reviewed the data and corrected the genome sizes to ensure consistency across the Abstract, Results section, and Table 1. We are deeply grateful for your careful reading and for bringing this mistake to our attention, which has significantly improved the accuracy and reliability of the manuscript.

 2.Astaxanthin Yield Comparison: The discussion rightly notes that the high astaxanthin yield of *R. paludigena* LL69-1 (1.186mg/L) is superior to that of "other microorganisms". However, the paper later cites *Rhodotorula* sp. CP72-2 (4.13mg/L) and *R. paludigena* SP9-15 (6.67mg/L) rephrase the statement on superiority to be more precise, perhaps by comparing it only to unoptimized strains or stating it is the highest yield in this study under the tested conditions, to avoid contradicting the later discussion. The final conclusion that optimizing conditions is necessary is appropriate.

 Answer: Thank you for your comment. We have revised the Discussion to clarify that *R. paludigena* LL69-1 showed the highest astaxanthin yield among the strains examined in this study under non-optimized conditions, rather than claiming overall superiority over other microorganisms. The text has been rephrased to clearly distinguish our results from reports of higher yields obtained under optimized or genetically modified conditions.

 3.Clarity on Strain Selection: In the Abstract, it is stated that HL26-1 had the greatest content and yield among *R. mucilaginosa* strains, and LL69-1 had the greatest content and yield among *R. paludigena* strains. While clear, you may consider clarifying in the Methods why only two strains ( *R. mucilaginosa* HL26-1 and *R. paludigena* LL69-1) were chosen for the full whole-genome sequencing and analysis (presumably because they were the best producers of their respective species).

 Answer: We thank the reviewer for this valuable comment. The criteria employed for selecting the strain for subsequent investigation have been described in the materials and methods section.

 4.Distinction between Carotenoids and Astaxanthin: The paper must consistently and clearly differentiate between Total Carotenoids and Astaxanthin. In the Results and Discussion, ensure that when values are presented, it is clear whether they refer to the total pool of pigments (carotenes, torulene, etc.) or specifically to the astaxanthin fraction, which was isolated and quantified by HPLC. The difference is vital for biotechnological assessment.

 Answer: We thank the reviewer for this important comment, and we revised as your suggestion. We would like to clarify that astaxanthin was quantified spectrophotometrically at a specific wavelength selected to minimize interference from other carotenoids, following the method reported by Li et al. (2012). In that study, absorbance at 530 nm showed a strong linear correlation with astaxanthin content.

 Accordingly, in this study, spectrophotometric measurements performed at the specified wavelength were used for astaxanthin quantification rather than total carotenoid determination. In addition, astaxanthin was independently identified and confirmed by HPLC based on retention time comparison and spiking experiments. We have revised the Results and Discussion sections to more clearly distinguish between total carotenoids and astaxanthin content wherever values are reported.

 Reference:

        - Li, Y., Miao, F., Geng, Y., Lu, D., Zhang, C., & Zeng, M. (2012). Accurate quantification of astaxanthin from Haematococcus crude extract spectrophotometrically. Chinese Journal of Oceanology and Limnology, 30(4), 627-637.

 5.HPLC: In the methods section, add chromatogram specifications, wavelength, flow rate, and injection volume.

 Answer: We thank the reviewer for this comment. Detailed HPLC chromatographic conditions, including column specifications, detection wavelength, flow rate, and injection volume, have now been added to the Methods section.

 6.TLC: TLC Rf value identical for all strains is suspicious—discuss variability.

 Answer: We thank the reviewer for this insightful comment. TLC was employed as a qualitative screening tool to indicate the presence of astaxanthin rather than for quantitative comparison. Although minor experimental variability in Rf values can occur due to factors such as solvent composition, plate condition, and development distance, the astaxanthin bands from all strains migrated within the same narrow *R _f_* range and corresponded to the astaxanthin standard under identical TLC conditions. For clarity, the manuscript has been revised to emphasize that TLC was used for qualitative identification, spectrophotometric analysis was used for astaxanthin quantification, and HPLC was employed to confirm the identity of astaxanthin based on retention time comparison and spiking experiments.

 7.Astaxanthin standard: Specify manufacturer purity in Methods.

 Answer: Specify manufacturer purity was added in the Methods section as your suggestion.

 8.Statistical Analysis Missing: Spectrophotometric data show mean ± SD, but no statistical tests are described. Mention statistical methods used to compare strains and add p-values where necessary.

 Answer: We thank the reviewer for this valuable comment. Statistical analysis using one-way ANOVA with a significance level set at *p* < 0.05 was conducted and mentioned in the Methods section and Fig. 2 description.

 Sincerely,

 The Authors

# Reviewer report: Reviewer response for version 1

**DOI:** 10.5256/f1000research.181137.r434447

**Published:** 2025-12-24

**Authors:** ANANDA NANJUNDASWAMY

**Affiliations:** 1 South Dakota State University, Brookings, South Dakota, USA

This is an excellent and timely study. With the growing global emphasis on developing natural sources of carotenoids, yeast-based production systems remain highly promising. The authors have evaluated strains of *Rhodotorula* spp. for the expression of astaxanthin biosynthetic genes, and the molecular tools employed are appropriate for the study. The biochemical analyses used to confirm astaxanthin production—such as TLC and HPLC—are well-suited and strengthen the conclusions.

 However, a few minor but important details need to be addressed before publication. The manuscript should clearly specify the type of statistical analyses used to evaluate the biochemical data. Additionally, the number of replicates used in the fermentation experiments must be provided. As currently presented, it appears that only a single replicate was used, which is not statistically acceptable. If biological or technical replicates were indeed included, please ensure that this information is explicitly stated.

 Overall, the manuscript contains valuable scientific information and, with these clarifications, would be suitable for publication.

Is the work clearly and accurately presented and does it cite the current literature?

Yes

If applicable, is the statistical analysis and its interpretation appropriate?

Yes

Are all the source data underlying the results available to ensure full reproducibility?

Yes

Is the study design appropriate and is the work technically sound?

Yes

Are the conclusions drawn adequately supported by the results?

Yes

Are sufficient details of methods and analysis provided to allow replication by others?

Yes

Reviewer Expertise:

Carotenoids, Bioprocessing, Pigmented yeasts, genetic engineering

I confirm that I have read this submission and believe that I have an appropriate level of expertise to confirm that it is of an acceptable scientific standard.

# Response

**Published:** 2025-12-30

**Authors:** Somboon Tanasupawat

**Affiliations:** Biochemistry and Microbiology, Chulalongkorn University, Bangkok, Thailand

Dear reviewer,

 We sincerely thank the reviewer for these valuable comments. The suggestions provided have been carefully considered and will be incorporated into the revised version of the manuscript.

 We have revised the manuscript to clearly specify the statistical analyses and experimental details used for evaluating the biochemical data. Astaxanthin content was quantified spectrophotometrically by measuring absorbance at the characteristic wavelength of astaxanthin (λ = 530 nm) using a calibration curve constructed from standard astaxanthin. The astaxanthin content and yield are now expressed as µg/g DCW and mg/L, respectively.

 We have also clarified the number of replicates used in the fermentation experiments. All experiments were performed in triplicate, and the results are reported as mean ± standard deviation. Statistical analysis was conducted using one-way ANOVA, with the significance level set at *p* < 0.05. These details have been explicitly added to the Materials and Methods section.

 Sincerely,

 The Authors

# Reviewer report: Reviewer response for version 1

**DOI:** 10.5256/f1000research.181137.r436642

**Published:** 2025-12-23

**Authors:** Kizhakkeyveetil Abdul Saleem Nimsi

**Affiliations:** 1 BIOSCIENCES, MES College Marampally (Ringgold ID: 210878), Aluva, Kerala, India

**Comments to the editor**

 The study has good potential and demonstrates substantial effort, but several important weaknesses need to be addressed before it can be considered for Indexing. Although the introduction is scientifically rich and relevant, it requires better organization, clearer presentation of the research gap, and improved readability to help readers fully grasp the study’s significance.

 The manuscript is structured into four sections, beginning with the isolation of pigmented yeasts; however, the source of samples, details of sample collection, and specific isolation methods are not described. In the screening phase, the procedures for purification steps, statistical validation, and criteria for significance, are absent. While the study focuses on two yeast strains ( *Rhodotorula mucilaginosa* HL26-1 and *Rhodotorula paludigena* LL69-1, the basis for selecting these strains is not explained in the Materials and Methods section.

 Overall, the Methods section is too brief, lacking essential details such as experimental design, number of replicates, appropriate controls, and how measurements were collected. Additional concerns include inconsistent use of units, typographical and formatting errors, repeated media compositions, and inconsistent use of abbreviations. The term “clonality” is inappropriately used in the preliminary selection stage and should be revised.

 Addressing these issues will significantly strengthen the manuscript by improving transparency, reproducibility, and scientific clarity.

 Comment 1: In the introduction author shall also discuss briefly about the pigmented yeasts in flower, their role, and importance.

 Comment 2: Author shall also write about the genes of pigmented yeast discovered so far and their importance.

 Comment 3: Media composition can write in bracket. Also, it is repeating in text.

 Comment 4: go through the manuscript, avoid typological error (e.g. Axs, Ø).

 Comment 5: uniform units throughout the manuscript (e.g. H, hour)

 Comment 6: sample collection, and processing are missing from materials and methods.

 Comment 7: Quantify the pigment and statistically validate the result.

 Comment 7: how can you identify the pigment is pure?

 Comment 8: is only one pigment is identified by TLC?

 Comment 9: describe the mobile phase in TLC.

 Comment10: how the astaxanthin purify for HPLC?

 comment: 11: include more reference after 2020. regarding pigmented yeasts from flowers.

Is the work clearly and accurately presented and does it cite the current literature?

Partly

If applicable, is the statistical analysis and its interpretation appropriate?

Partly

Are all the source data underlying the results available to ensure full reproducibility?

Yes

Is the study design appropriate and is the work technically sound?

Partly

Are the conclusions drawn adequately supported by the results?

Yes

Are sufficient details of methods and analysis provided to allow replication by others?

Yes

Reviewer Expertise:

Diversity yeasts, pigments, plant microbe interaction, metagenomics

I confirm that I have read this submission and believe that I have an appropriate level of expertise to confirm that it is of an acceptable scientific standard, however I have significant reservations, as outlined above.

# Response

**Published:** 2025-12-30

**Authors:** Somboon Tanasupawat

**Affiliations:** Biochemistry and Microbiology, Chulalongkorn University, Bangkok, Thailand

Dear reviewer

 We sincerely thank the reviewer for these valuable comments. The suggestions provided have been carefully considered and will be incorporated into the revised version of the manuscript.

 The introduction has been revised to improve organization, readability, and logical flow. The research gap and the significance of the study are now stated more clearly to highlight the novelty of the work.

 The Materials and Methods section has been greatly expanded. The source and details of sample collection were described. The specific method for yeast isolation was added. In addition, the measurement methods, and data analysis procedures have been clarified to enhance transparency and reproducibility. Inconsistencies in units, typographical and formatting errors, repeated media descriptions, and inconsistent use of abbreviations have been corrected. The inappropriate use of the term “clonality” has been revised.

 A detailed point-by-point response addressing each of the reviewer’s comments is provided below.

 Comment 1: In the introduction author shall also discuss briefly about the pigmented yeasts in flower, their role, and importance.

 Answer: We thank the reviewer for this comment. A brief background about role and importance of the pigmented yeasts in flowers has been described in the last paragraph of the introduction part.

 Comment 2: Author shall also write about the genes of pigmented yeast discovered so far and their importance.

 Answer: We thank the reviewer for this comment. We would like to clarify that we have indeed discussed in the introduction part about the genes associated with pigmented yeasts, particularly those in the genus *Phaffia*, highlighting their significance mechanism in astaxanthin synthesis. However, concerning the genes of pigmented yeasts in the genus *Rhodotorula*, we regret to inform you that we cannot provide a detailed report on their expression at this time. Currently, there is a lack of clear studies documenting the expression of these genes in *Rhodotorula *species. This limitation has prevented us from including specific information in our manuscript.

 Comment 3: Media composition can write in bracket. Also, it is repeating in text.

 Answer: Media composition was written in bracket and the repeating text was edited.

 Comment 4: go through the manuscript, avoid typological error (e.g. Axs, Ø).

 Answer: The typographical errors were edited.

 Comment 5: uniform units throughout the manuscript (e.g. H, hour)

 Answer: The unit “h” throughout the manuscript was edited to be hours.

 Comment 6: sample collection, and processing are missing from materials and methods.

 Answer: The specifics of sample collection and processing were described.

 Comment 7: Quantify the pigment and statistically validate the result.

 Answer: The pigment content was quantified spectrophotometrically by measuring absorbance at the characteristic wavelength of astaxanthin (λ = 530 nm) using a calibration curve constructed from standard astaxanthin. The pigment concentration was expressed as mg/L (or µg/g DCW). All experiments were performed in triplicate, and results were reported as mean ± standard deviation. Statistical analysis was conducted using one-way ANOVA with a significance level set at *p* < 0.05.

 Comment 7: how can you identify the pigment is pure?

 Answer: We thank the reviewer for this valuable comment. We would like to clarify that the pigment was not subjected to additional purification prior to analysis. Astaxanthin was identified by HPLC based on comparison of its retention time with that of an authentic astaxanthin standard. In addition, a spiking experiment was performed by adding the astaxanthin standard to the sample extract, which resulted in a proportional increase in the target peak without the appearance of additional peaks. The HPLC chromatograms of the standard, sample, and spiked sample are provided in Supplementary Figure 1, which together confirm the identity of astaxanthin in the extract.

 Comment 8: is only one pigment is identified by TLC?

 Answer: We thank the reviewer for this important comment. TLC analysis revealed the presence of multiple pigment bands, indicating the existence of several carotenoid components in the extract. However, astaxanthin was identified based on the comparison of its *R _f_* value with that of the astaxanthin standard analyzed under the same chromatographic conditions. The band corresponding to astaxanthin showed a matching *R _f_* value and similar coloration to the standard, confirming its identity.

 Comment 9: describe the mobile phase in TLC.

 Answer: We thank the reviewer for this comment. The mobile phase used for TLC analysis was a mixture of acetone and hexane at a ratio of 1:3 (v/v). This solvent system enabled effective separation of carotenoid pigments, and astaxanthin was identified by comparing the *R _f_* value and coloration of the sample band with those of the astaxanthin standard analyzed under identical conditions. The description of the TLC mobile phase has been added to the Methods section.

 Comment10: how the astaxanthin purify for HPLC?

 Answer: We thank the reviewer for this comment. We would like to clarify that astaxanthin was not subjected to a separate purification step prior to HPLC analysis. Instead, astaxanthin was extracted from yeast cells using DMSO, followed by centrifugation and filtration to remove cell debris. The clarified extract was directly analyzed by HPLC. Astaxanthin was identified based on comparison of its retention time with an authentic astaxanthin standard, and further confirmed by spiking experiments. This clarification has been added to the revised manuscript.

 comment: 11: include more reference after 2020. regarding pigmented yeasts from flowers.

 Answer: An update report on pigmented yeast isolated from flowers was presented as your suggestion.

 Sincerely,

 The Authors
